# Bead-based approaches for increased sensitivity and multiplexing of CRISPR diagnostics

**DOI:** 10.1038/s41551-025-01498-2

**Published:** 2025-09-22

**Authors:** Sameed M. Siddiqui, Nicole L. Welch, Tien G. Nguyen, Amaya Razmi, Tianyi Chang, Rebecca Senft, Jon Arizti-Sanz, Marzieh E. Mirhashemi, David R. Stirling, Cheri M. Ackerman, Beth A. Cimini, Paul C. Blainey, Pardis C. Sabeti, Cameron Myhrvold

**Affiliations:** 1 Broad Institute of Massachusetts Institute of Technology (MIT) and Harvard, Cambridge, MA USA; 2 Computational and Systems Biology, Massachusetts Institute of Technology, Cambridge, MA USA; 3 Harvard Program in Virology, Division of Medical Sciences, Harvard Medical School, Boston, MA USA; 4 Department of Molecular and Cellular Biology, Harvard University, Cambridge, MA USA; 5 Harvard-MIT Program in Health Sciences and Technology, Cambridge, MA USA; 6 Department of Biological Engineering, Massachusetts Institute of Technology, Cambridge, MA USA; 7 Koch Institute, , Massachusetts Institute of Technology, Cambridge, MA USA; 8 Howard Hughes Medical Institute, Chevy Chase, MD USA; 9 Department of Organismic and Evolutionary Biology, Harvard University, Cambridge, MA USA; 10 Department of Immunology and Infectious Diseases, Harvard T.H. Chan School of Public Health, Harvard University, Boston, MA USA; 11 Department of Medicine, Division of Infectious Diseases, Massachusetts General Hospital, Boston, MA USA; 12 Massachusetts Consortium on Pathogen Readiness, Boston, MA USA; 13 Department of Molecular Biology, Princeton University, Princeton, NJ USA; 14 Omenn-Darling Bioengineering Institute, Princeton University, Princeton, NJ USA; 15 Department of Chemical and Biological Engineering, Princeton University, Princeton, NJ USA; 16 Department of Chemistry, Princeton University, Princeton, NJ USA

**Keywords:** Assay systems, Biotechnology, Biomedical engineering

## Abstract

CRISPR-based diagnostics have emerged as a promising tool for fast, accurate and portable pathogen detection. There has been rapid progress in pre-amplification processes and CRISPR-related enzymes used in these approaches, but the development of reporter systems and reaction platforms has lagged behind. In this paper, we develop bead-based techniques to address these gaps. First, we develop a novel bead-based split-luciferase reporter system with up to 20× sensitivity compared with standard fluorescence-based reporter design in CRISPR diagnostics. Second, we develop a highly deployable, bead-based platform capable of detecting nine distinct viral targets in parallelized, droplet-based reactions, with sensitivity reaching as low as 2.5 copies per µl of input RNA. We demonstrate the enhanced performance of both approaches on synthetic and clinical sample sensitivity, speed, multiplexing and deployability.

## Abstract (web summary)

Two bead-based approaches for CRISPR diagnostics increase sensitivity and deployability in resource-constrained settings.

## Main

The COVID-19 pandemic has highlighted the need for rapidly deployable diagnostic technologies able to respond to new pathogens and emergent variants anywhere in the world^1^. However, no current technology uniquely meets the sensitivity, specificity, deployability, speed and multiplexing needs required for a broad and robust response to infectious disease outbreaks. Quantitative polymerase chain reaction (qPCR), widely considered a gold standard due to its sensitivity and specificity, cannot be deployed readily and remains limited in multiplexing ability^2–4^. Next generation sequencing (NGS) is similarly sensitive, specific, and able to detect many pathogens and variants; however, it is expensive, has a long turnaround time, and requires substantial technical expertise to deploy for viral surveillance^5^. On the other hand, antigen capture tests are readily deployable and affordable, but are less sensitive and specific than nucleic acid tests and are not rapidly adaptable for new pathogens^6,7^.

CRISPR-based systems offer an alternative approach that is well-poised to address pathogen diagnostic needs. Specifically, CRISPR effectors Cas12 and Cas13 exhibit collateral cleavage activity upon recognition of their target DNA or RNA, respectively, enabling these enzymes to act as target-specific sensors^1,8–10^. Since their introduction, there has been substantial work in developing CRISPR-based diagnostic systems, with assays for diverse pathogens such as influenza, Zika virus and SARS-CoV-2 developed across different combinations of CRISPR effectors, amplification modalities, imaging tools and reaction platforms^1,11–13^. Much of this effort has been limited to fluorescence and lateral flow strip readouts, leaving room for orthogonal advances in reporter design and reaction barcoding to improve deployability, sensitivity and multiplexing capability^1,6,14–18^.

Bead-based systems, such as AlphaLISA^19,20^, have led to recent advances in protein detection due to their ability to compartmentalize reaction components and may serve as a basis to advance CRISPR-based nucleic acid detection. For example, separating reaction components onto different bead types could create a highly sensitive split-luciferase reporter for Cas13 diagnostics. Separately for multiplex detection, Luminex consists of fluorescently colour-coded beads that are coupled to different antibodies, enabling pooled identification of separate targets in a single reaction^21,22^. This technology suggests that colour-coded beads could lend themselves well to having different targets for Cas13 detection on each bead. We explored both technological approaches to expand the breadth of CRISPR-based diagnostic platforms.

We first considered how bead-based readouts could improve sensitivity in point-of-need CRISPR-based diagnostic assays. When a sample is used as input in CRISPR-Cas13 diagnostic assays, the target genetic material is first amplified using amplification methods such as quantitative reverse transcription PCR (RT–qPCR) (typically involving 30–40 cycles) or recombinase polymerase amplification (RPA), either before or in the same reaction as Cas13 detection. Assays such as Streamlined Highlighting of Infections to Navigate Epidemics (SHINE), which couple isothermal amplification with Cas13 detection in the same reaction (‘one-pot’) are well-suited for point-of-need deployment due to their ease of use^6,18^. These assays have traditionally used fluorescence-based reporters, primarily consisting of a fluorescein (FAM) dye linked by a short oligonucleotide sequence to a quencher^1,6,8,14^. While these assays have performed well, fluorescence-based technologies are known to have high background signal and low sensitivity compared with bioluminescence technologies^6,23^.

A bead-based luminescent split reporter system which links nucleic acid detection with nanoluciferase (NanoLuc) complementation could provide an attractive alternative to fluorescent reporters, enabling rapid attomolar detection with a high dynamic range^24–26^. A two-bead system with a large protein subunit (LgBiT) and a smaller peptide subunit (HiBiT) each coupled to a separate bead type may serve as basis for a Cas13 cleavage reporter if at least one of the protein subunits is coupled via a Cas13-cleavable RNA linker. By virtue of being coupled to separate beads, LgBiT and HiBiT can be largely separated from each other and kept catalytically inactive. In the presence of the target, Cas13 collateral cleavage of the bead RNA linkers could reverse this separation and allow the formation of complemented NanoLuc. Therefore, for point-of-need use, a new split-luciferase-based reporter system could be well-poised to improve sensitivity while simultaneously removing the requirement of a light source as in fluorescence-based systems.

We next considered how a bead-based system could improve multiplexed diagnostic testing at point of care. We previously developed the CRISPR-based Combinatorial Arrayed Reactions for Multiplexed Evaluation of Nucleic acids (CARMEN) and microfluidic CARMEN (mCARMEN)^16,17^, and demonstrated their multiplexing and sensitivity across samples and pathogens. These platforms, however, require high technical expertise and costly equipment to achieve sample or patient barcoding, restricting their deployability in resource-limited settings^16,17^. This constraint leaves an opportunity to replace barcoding with a less resource-intensive, bead-based approach.

A colour-coded bead-based approach that couples beads to distinct CRISPR RNAs (crRNAs) could be used to create a localized separation of crRNA, enabling an assay with multiplexed nucleic acid targets. Given previous work in microparticle-based dropletization, such colour-coded beads could allow equipment-free nanolitre droplet generation^27^, with each droplet containing Cas13 detection master mix and approximately one colour-coded crRNA bead. With crRNA-specific detection reactions occurring in parallel across different droplets, this could in turn enable a highly parallelized reaction which could be imaged in a fluorescence microscope or imaging plate reader to determine bead target-specific detection. Thus, for point-of-care use, a bead-based, low-cost platform capable of parallelized, dropletized detection of multiple targets may enable a sensitive, robust and highly multiplexed solution for resource-limited settings.

Here we explore the applicability of bead-based approaches to increasing sensitivity, multiplexing and deployability in CRISPR diagnostics through two distinct technologies. First, to advance CRISPR diagnostics towards improved point-of-need accessibility, we designed a bead-based split-luciferase reporter (bbLuc) and examined this readout modality in an amplification-free reaction and in the amplification-coupled SHINE diagnostic platform. Next, for laboratory and point-of-care settings, we developed and validated a bead-based deployable multiplexed diagnostics platform (bbCARMEN) and further investigated its performance in an implementation of a panel of nine respiratory viruses^16^.

## Results

### Coupling Cas13 activity to a split nanoluciferase readout

**Fig. 1:**
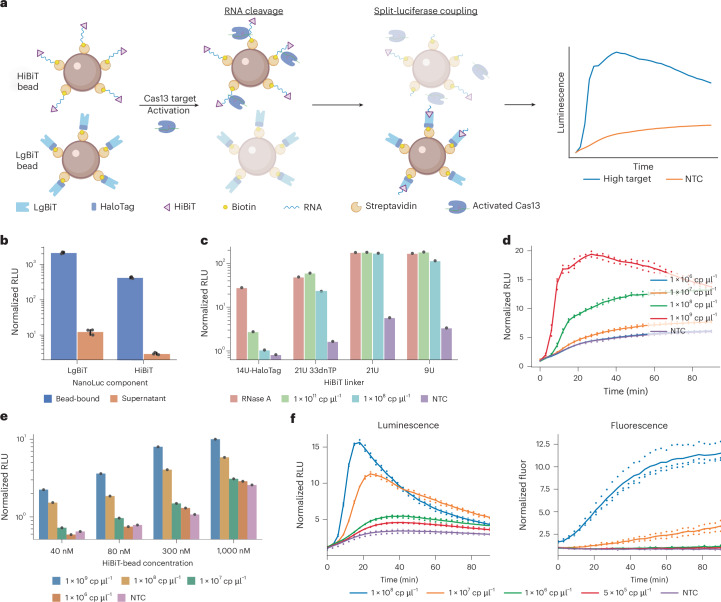
Initial design and optimization for luminescent reporters. **a**, Schematic of the split-luciferase reporter system. HiBiT and LgBiT are initially separated on different beads. Cas13 cleavage of the RNA linker releases HiBiT, allowing it to bind to LgBiT and reconstitute active luciferase. **b**, Beads bound with 80 nM HiBiT or 80 nM LgBiT show durable coupling through storage and wash cycles. Luminescence measured in relative luminescence units (RLU). **c**, Single-bead luminescence assay to compare different RNA linkers used to couple HiBiT to beads (21U 33dnTP HaloLigand-HaloTag-based, 21U SPAAC-based, 9U SPAAC-based linkers). RNase A or Cas13/target mixes were added to coupled beads to enable cleavage, with the resulting cleaved products separated from the beads magnetically and added to LgBiT in solution. **d**, Luminescence kinetics of full assay, before bead optimization (80 nM HiBiT, 80 nM LgBiT). The normalized signal is lower than in previous reactions due to differences in on-bead versus in-solution reaction kinetics. Luminescence values normalized to the average no template control (NTC) signal at the first-collection timepoint. **e**, Optimization of HiBiT bead concentrations in amplification-free luminescence assay, with LgBiT concentration maintained at 80 nM. **f**, Optimized luminescence amplification-free assay kinetics compared to fluorescence assay on varied synthetic RNA targets, 90 min (80 nM LgBiT, 300 nM HiBiT, 200 μM furimazine).

We developed a bead-based luciferase reporter (bbLuc) to couple Cas13 activity with a split nanoluciferase-based readout (Fig. 1a). As a first step, we probed whether it is possible to couple HiBiT and LgBIT to beads using a Cas13-cleavable RNA-based linker. We attached HiBiT and LgBiT to biotinylated oligonucleotides using HaloLigand-HaloTag-based covalent linking, thereby enabling coupling to streptavidin-coated beads (Fig. 1b)^28^. Notably, this coupling enabled target-mediated Cas13 cleavage of HiBiT-linked nanoparticles (Fig. 1c). However, we did not observe Cas13 cleavage of LgBiT-linked nanoparticles, most probably due to increased steric hindrance of Cas13 cleavage in proximity with the larger LgBiT enzyme compared with the HiBiT peptide. As such, we focused our subsequent design efforts on cleavable HiBiT nanoparticles.

Having demonstrated the ability to couple HiBiT to beads using a Cas13-cleavable RNA-based linker, we next designed improved linkers to optimize and enable more efficient cleavage. We found through a side-by-side comparison of Cas13-based and RNase A-based cleavage that the initial Cas13 cleavage of HaloTag-based linkers was inefficient (Fig. 1c). We hypothesized that this inefficiency may have been caused again by steric hindrance between the beads and the HaloTag-HiBiT complex, reducing accessibility of Cas13 to cleavage sites. We therefore increased the linker length (Supplementary Table 1) and changed the linkage chemistry by using strain-promoted azide–alkyne click chemistry reaction (SPAAC) to connect the HiBiT peptide to the oligonucleotide linker. This design enabled substantially more efficient Cas13 cleavage compared with the HaloTag-HiBiT designs, showing equivalent cleavage of the HiBiT linker from Cas13 and RNase A (Fig. 1c).

Next, we characterized and further optimized the performance of an amplification-free assay with both HiBiT and LgBiT beads in solution. Using 80 nM HiBiT peptide coupled to nanoparticles in solution, we were able to achieve detection down to 10^7^ copies per µl of input RNA with an unoptimized design (Fig. 1d). While this was similar to a fluorescence-based amplification-free assay, we observed further improvements in sensitivity when the surface density of HiBiT peptides on nanoparticles was increased to a concentration of 300 nM (Fig. 1e). HiBiT concentrations above 300 nM and LgBiT concentrations above 80 nM resulted in more inconsistency and lower sensitivity (Supplementary Fig. 2), possibly due to an increased viscosity of the solution caused by a higher peptidic charge on the beads.

We compared the performance of bbLuc to a conventional fluorescent reporter in the amplification-free assay. Within 60 min, our luminescent reporter detected down to ~5 × 10^5^ copies per µl of the input target compared with 1 × 10^7^ copies per µl for the fluorescent reporter. We were thus able to achieve a 20× increase in sensitivity using the luminescent reporter within 90 min (Fig. 1f and Supplementary Fig. 3).

### Integration of luminescent reporter into SHINE

**Fig. 2:**
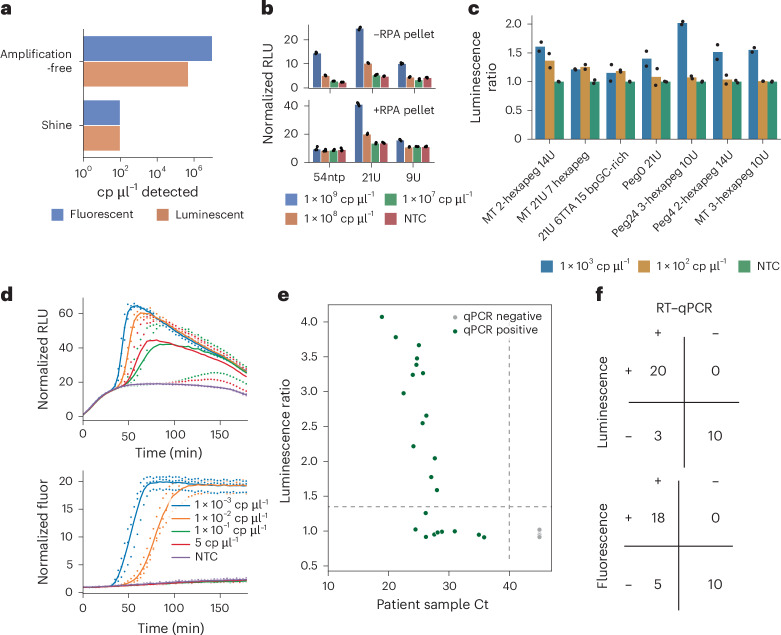
bbLuc integration with SHINE. **a**, Luminescence comparison of SHINE and amplification-free systems before optimization; initial luminescence comparison of SHINE and amplification-free systems. Without optimization, the 20× sensitivity advantage of bbLuc in the amplification-free setting was not maintained in SHINE. **b**, In amplification-free experiments across 3 different luminescent bead linker types (54 ntp DNA–RNA linkers, 21 uracil linkers and 9 uracil linkers), different linker types are affected differentially by the addition of recombinase polymerase amplification (RPA) reagents, necessary for downstream SHINE. In particular our original linker with 54 ntps, used in Figs. 1d–f and 2a, showed reduced Cas13-based cleavage and signal (data shown after 26 min) **c**, Luminescence SHINE performance of 7 tested RNA linkers after 3 h (all 21 linker types tested shown in Supplementary Fig. 6) **d**, Final optimized reaction kinetics with bbLuc SHINE (80 nM LgBiT, 300 nM HiBiT, 2-hexapeg 14U linker, 150 µM furimazine, 60 nM RPA primers). **e**, SARS-CoV-2 detection using bbLuc with RNA from patient samples, displayed as the ratio of NTC to patient sample bbLuc signal at 90 min. Threshold line plotted as 1.35, determined empirically. **f**, Confusion matrices of SARS-CoV-2 detection in patient samples using bbLuc SHINE and fluorescence SHINE compared to side-by-side RT–qPCR results. Ct values and luminescence ratios in **e** computed as means of three technical replicates. Positive versus negative readouts as determined by three technical replicates for fluorescent SHINE, bbLuc SHINE and qPCR.

Having optimized bbLuc in an amplification-free setting, we then assessed its performance in the one-pot, amplification-coupled SHINE platform. At first, we found that the luminescent reporter did not perform better than the conventional fluorescent reporter in the SHINE setting, compared with the 20× enhancement we achieved in the amplification-free setting (Fig. 2a). We hypothesized that the increased complexity of the SHINE reaction, compared with the amplification-free reactions, may have introduced inhibitory interactions between the bead complexes and the reaction components, leading to the observed decrease in relative sensitivity.

We explored the potential causes of reduced sensitivity of our luminescent reporter in the SHINE context by testing different concentrations of a molecular crowding reagent and assessing whether individually spiking in SHINE components to amplification-free reactions interfered with detection. We first examined possible molecular crowding effects caused by bead addition to the assay by modifying polyethylene glycol (PEG) concentration and molecular weight in the SHINE reaction buffer, but found that the PEG parameters in fluorescence SHINE were optimal with the luminescence system (Supplementary Fig. 4). We then probed possible inhibitory effects on the beads from the constituent SHINE proteins by individually spiking reaction components into separate amplification-free reactions, finding a 1,000-fold reduction in performance upon addition of RPA pellets (Fig. 2b and Supplementary Fig. 5). We found that this reduction in sensitivity was largely caused by single-stranded binding (SSB) protein reducing Cas13 cleavage of the beads (Supplementary Fig. 6).

On the basis of the observation that the SSB used in RPA was inhibiting our bead linkers, we tested a series of new linkers to find designs that improved detection in SHINE. We hypothesized that our original HiBiT-bead linker design consisting of 54 nucleotides (21 uracils and 33 dNTPs) may have served as a binding site for SSB, thereby sterically interfering with Cas13 cleavage of the linker. We first tested shorter linker designs without any dNTPs, finding reduced inhibition of detection by 21U and 9U linkers in the presence of RPA components compared with our original design (Fig. 2b). We went on to test 27 different linker designs (Fig. 2c, Supplementary Figs. 7 and 8, and Table 1), finding that a 2-hexapeg 14U linker provided the highest sensitivity among our reporters.

In our first set of optimizations to the luminescence SHINE assay, we varied the concentrations of furimazine, RPA primers and magnesium acetate, setting concentrations of 50 µM, 140 nM and 14 nM, respectively (Supplementary Figs. 9–11). We found that this initial version of the assay, bbLucV0 SHINE, had an analytical sensitivity of 32 copies per µl of input RNA in 75 min (Supplementary Fig. 12).

We compared bbLucV0 SHINE and fluorescence SHINE to the gold-standard RT–qPCR test for COVID-19. Out of 59 usable samples, bbLucV0 SHINE accurately identified 26 out of 29 positive cases (89.7%), while fluorescence SHINE only identified 23 out of 29 cases (79.3%) (Supplementary Fig. 13). Notably, bbLucV0 SHINE detected all the positive cases identified by fluorescence SHINE, plus three additional cases with low viral loads. Both methods correctly identified all negative cases. This demonstrates the superior sensitivity of bbLucV0 SHINE in detecting COVID-19, especially in cases with low viral loads. Critically, the luminescent reporter is used in SHINE as a drop-in replacement for fluorescent reporters, enabling enhanced sensitivity while still maintaining one-pot reaction chemistry.

Although dual bead systems can be prone to non-specific thermodynamic interactions, we found that careful optimization of linker design, conjugation chemistry and component concentrations led to increased Cas13-mediated cleavage signal (Supplementary Figs. 2, 4 and 7–11). These improvements enabled robust detection at low target input concentrations, including 100% specificity in patient samples (Supplementary Fig. 13), indicating clear separation between true signal and background noise.

### Single-timepoint luminescence readout in SHINE

To increase the deployability of bbLucV0 SHINE, we modified the reaction chemistry to enable a simplified readout process using a single-endpoint measurement instead of a multi-timepoint plate reader. We hypothesized that increasing the concentration of furimazine, the substrate for NanoLuc, would enable a longer-lasting luminescent signal, thereby allowing for a single-timepoint measurement on a portable luminometer. By increasing the furimazine concentration from 50 nM to 150 nM and delaying its addition to the master mix to prevent salting out, we extended the duration and sensitivity of the luminescent signal, enabling 20× sensitivity for luminescence SHINE compared with fluorescence SHINE (Fig. 2d). To confirm that the observed signal was due to specific Cas13 cleavage of our luciferase reporter, we performed a no-Cas13 control reaction, which showed no detectable signal above background (Supplementary Fig. 14), further validating the assay. We refer to this assay as bbLucV1 SHINE, or simply, bbLuc SHINE.

In clinical samples, the bbLuc SHINE method demonstrated exceptional accuracy in detecting both positive and negative cases within 90 min, outperforming the fluorescence SHINE method. The bbLuc SHINE method successfully identified 20 out of 23 positive cases and achieved a perfect 10 out of 10 accuracy in identifying negative cases (Fig. 2e,f). In contrast, the fluorescence SHINE method detected 18 out of 23 positive cases and also achieved a perfect 10 out of 10 accuracy in identifying negative cases (Fig. 2f).

**Fig. 3:**
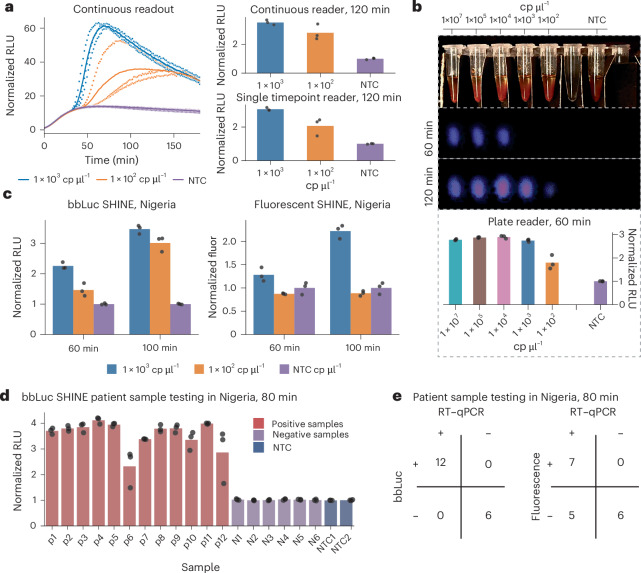
Optimizing bbLuc for deployability. **a**, Comparison of kinetic and single-timepoint measurements of bbLuc SHINE using synthetic SARS-CoV-2 RNA with a dilution series and NTC, demonstrating compatibility with both plate readers and portable luminometers. **b**, Demonstration of bbLuc SHINE’s sensitivity using cellphone-based imaging of synthetic SARS-CoV-2 RNA at 10^3^ copies per µl after 60 and 120 min incubation, with a positive signal visible to the naked eye in some instances. Side-by-side comparison with plate reader data shown at the bottom. **c**, Comparison of bbLuc and fluorescence SHINE assays in Nigeria using synthetic SARS-CoV-2 RNA, demonstrating improved sensitivity and speed of bbLuc SHINE versus fluorescence SHINE. **d**, Patient sample testing data in Nigeria, showing 80-min readout for bbLuc SHINE assay, with raw sample signal normalized by NTC value. **e**, Confusion matrices comparing bbLuc SHINE and fluorescence SHINE in Nigeria with 12 RT–qPCR-positive and 6 RT–qPCR-negative patient samples. Results shown for 80-min timepoints, with other timepoints shown in Supplementary Fig. 15. Positive versus negative readouts as determined by three technical replicates for fluorescent SHINE, bbLuc SHINE and qPCR.

The extended luminescence signal in bbLuc SHINE, compared with the rapid signal decay observed in bbLucv0 SHINE, eliminates the need for kinetic timepoints or continuous readout. This advancement enables the use of low-cost plate readers, which lack the built-in heating functionality of more expensive models, for accurate single-timepoint measurements. The robust luminescent signal also allows for detection using a portable, handheld luminometer with a single-timepoint reader instead of a continuous readout (Fig. 3a).

To push the boundaries of bbLuc’s field deployability, we explored both cellphone-based imaging and simplified sample processing. We were able to detect as few as 10^3^ copies per µl of SARS-CoV-2 RNA using only a smartphone camera for imaging (Fig. 3b). This signal was often visible to the naked eye, underscoring the assay’s exceptional sensitivity and potential for deployment in extremely resource-limited settings where specialized equipment may not be available. Furthermore, in controlled studies with synthetic targets, we evaluated bbLuc using the SHINE v2-validated extraction-free protocol with room-temperature viral lysis. bbLuc maintained robust detection capability in the presence of transport media and lysis buffer, suggesting potential for simplified diagnostic workflows in point-of-need settings (Supplementary Fig. 15).

### Enhanced sensitivity and field validation in Nigeria

To assess the performance of our optimized bbLuc assay in a low-resource country, we conducted a field validation study in Nigeria. We first performed a dilution series of synthetic SARS-CoV-2 RNA and observed a 10-fold increase in sensitivity for the SHINE reaction within 75 min when using the luminescent bbLuc reporter compared with the traditional fluorescent reporter (Fig. 3c), consistent with our earlier experiments. Next, we tested 18 patient samples (12 RT–qPCR-positive and 6 RT–qPCR-negative) using both the luminescence and fluorescence SHINE assays. The luminescence assay correctly identified all 12 positive samples within 60 min (Fig. 3d), compared with 3 out of 12 samples within 60 min, 7 out of 12 samples within 80 min, and 9 out of 12 samples after 3 h with fluorescence (Fig. 3e and Supplementary Fig. 16). All RT–qPCR-negative samples were negative by bbLuc; however, one replicate of a negative sample in fluorescence SHINE displayed a false-positive after ~100 min, potentially indicating a contamination or other technical error in that replicate. In aggregate, the improved speed and sensitivity shown in these results highlight the strengths of luminescence assay, particularly useful for deployable, point-of-need diagnostic modalities.

### Equipment-free bead-based droplet generation for multiplexed fluorescent Cas13 detection

**Fig. 4:**
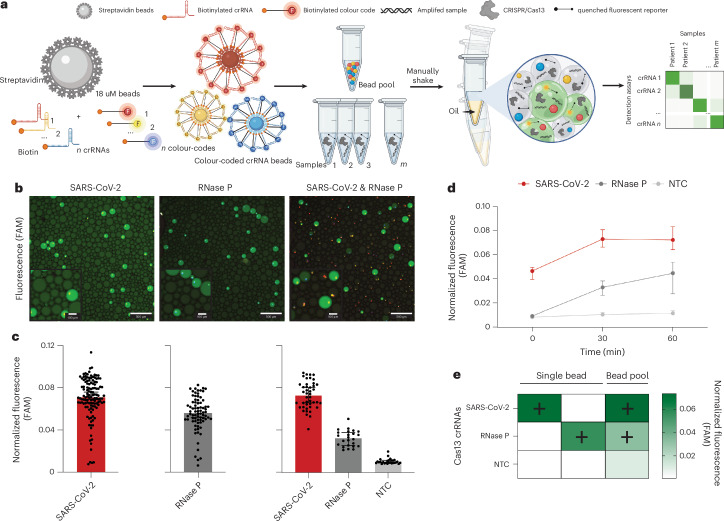
Development of bbCARMEN with biotinylated crRNA beads in equipment-free generated droplets. **a**, Schematic of the bbCARMEN workflow. **b**, Merged fluorescence images of colour-coded crRNA beads in droplets. SARS-CoV-2: AF546 shown as yellow; RNase P: AF647 shown as blue; FAM reporter shown as green. **c**, Fluorescence from droplets from individual crRNA bead solutions and pooled bead solutions at 30 min post-reaction initiation. Individual points represent fluorescence from individual droplets. Median fluorescence values are plotted with 95% CIs. **d**, Fluorescence kinetics of amplified SARS-CoV-2 synthetic gene fragment at 10^6^ copies per μl and RNase P in pooled crRNA bead solution from **b** and **c**. Red, SARS-CoV-2; dark grey, RNase P; light grey, NTC. Median fluorescence values are plotted with 95% CIs. **e**, Heat map showing SARS-CoV-2, RNase P and NTC median fluorescence values at 30 min post-reaction initiation in single and pooled crRNA bead solutions from **b** and **c**, conducted at 10^6^ copies per μl.

Next, we developed a bead-based approach to reduce cost and increase deployability of CRISPR-based multiplexed testing for laboratory and point-of-care settings. To enable bead-based multiplexing of target detection, we first attached target-specific biotinylated crRNAs to colour-coded, streptavidin-coated beads, examining results when crRNA was attached to beads via either 3’ end biotinylation or 5’ end biotinylation (Fig. 4a and Supplementary Fig. 17). With 3’ end biotinylation, we found considerable Cas13 cleavage in target compared with no target control (NTC) down to 1 copy per μl of input. In comparison, with 5’ end biotinylation, Cas13 cleavage was not able to distinguish an input of 10^4^ copies per µl from NTC (Supplementary Fig. 18). As such, we used 3’ biotinylated crRNA beads moving forward.

**Fig. 5:**
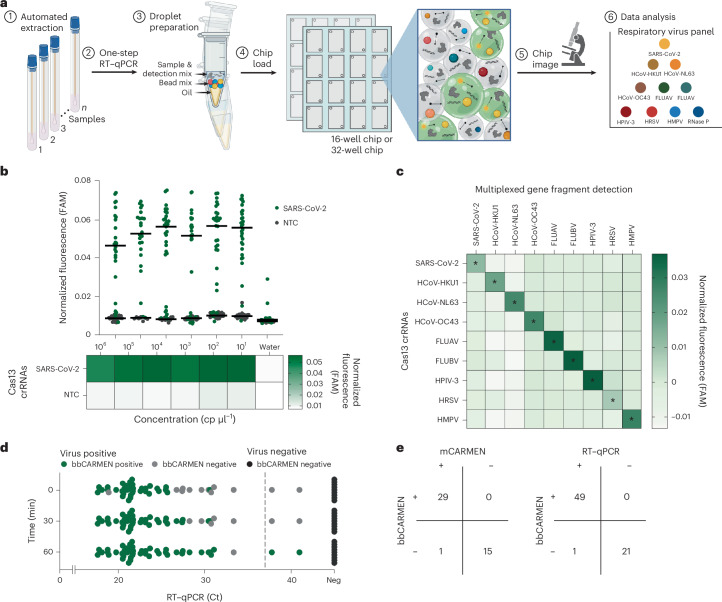
Implementation of respiratory virus panel on bbCARMEN. **a**, Schematic of multiplexed RVP assay with each of the 9 viruses on panel differentiated by distinct colour codes. **b**, Fluorescence across SARS-CoV-2 dilution series from 10^6^–10^1^ copies per μl and corresponding NTC fluorescence in a pooled bead solution containing all 10 RVP crRNAs. Green, SARS-CoV-2; grey, NTC. Line indicates median fluorescence 30 min post-reaction initiation. **c**, Fluorescence at 10^4^ copies per μl for all 9 viruses on RVP. Fluorescence shown as the median value across 20 replicates at 30 min post-reaction initiation. *, expected reactivity based on crRNA sequence design. **d**, Scatterplot comparison of SARS-CoV-detection at 0-, 30- and 60-min timepoints using bbCARMEN and RT–qPCR. Ct values computed as means of three technical replicates. **e**, Concordance of RT–qPCR and NGS results compared to CARMEN v2. Right: RT–qPCR results. Left: NGS results. Positive versus negative ground truth as determined by three technical replicates for qPCR.

To show that our bead-based CARMEN (bbCARMEN) approach would work in the context of multiple targets, we developed assays for SARS-CoV-2 and a human internal control (RNAse P) both individually and in combination (Fig. 4b–e and Supplementary Fig. 19). We added colour-coded beads to a solution containing Cas13 and additional detection components with amplified patient samples. For each reaction, we combined the detection master mix with oil, shaking to form miniature droplets containing the master mix and one colour-coded bead (Fig. 4b and Supplementary Fig. 20). We then loaded samples on custom, prefabricated flow cells for readout using fluorescence microscopy and automated image analysis to track both the colour-coded crRNA beads and the signal from Cas13 activity (Figs. 4c,d and 5a, and Supplementary Figs. 17 and 18). By 30 min, we were successfully able to detect fluorescence intensity in droplets above background for all conditions (Fig. 4d,e and Supplementary Fig. 21).

### Respiratory virus panel implementation on bbCARMEN

We next tested the performance of a larger multiplexed bbCARMEN by implementing a 9-target respiratory virus panel (RVP) previously characterized on the mCARMEN platform (Fig. 5a)^16^. We conjugated human RNase P crRNA (as an internal control) and each crRNA from the RVP to beads with target-specific colour codes and added the beads to the reaction along with patient sample (Supplementary Fig. 20). We used fluorescence microscopy to track patient sample signals over time and map crRNA/colour-code combinations (Supplementary Fig. 20). With the RVP, bbCARMEN successfully distinguished all bead colour codes from one another across replicates, while simultaneously observing on-target signal with minimal to no off-target signal for each panel member (Fig. 5b,c and Supplementary Fig. 22).

We verified the accuracy of these results by conducting limit of detection (LOD) studies for all respiratory virus panel members (Fig. 5b, and Supplementary Figs. 20 and 21). Overall, we found the LOD for each virus (SARS-CoV-2 and HCoV-HKU1: 2.5 copies per µl, FLUBV and HPIV3: 5 copies per µl, FLUAV and HRSV: 10 copies per µl, HMPV: 20 copies per µl, HCoV-NL63 and HCoV-OC43: 40 copies per µl) to be in line with or slightly higher than the LOD of the RVP on mCARMEN.

To more rigorously validate sensitivity, we tested 47 freshly collected (Delta and Omicron) SARS-CoV-2 specimens and 9 negatives on the basis of RT–qPCR and NGS results (Fig. 5d,e). All but one positive specimen (97.9%) was deemed virus-positive by bbCARMEN within 60 min, and all negative samples were correctly deemed negative. To further assess the sensitivity of this assay, we also tested bbCARMEN on two cohorts of virus-positive samples previously characterized with mCARMEN and clinically validated comparator assays and stored at −80 °C (Supplementary Fig. 23). Of the 60 positive samples tested (30 SARS-CoV-2-positive and 30 HSRV-positive), bbCARMEN detected 56 (93.33%) within 60 min, exhibiting robust sensitivity even in samples subjected to a freeze–thaw cycle.

### Automated readout of bbCARMEN using commercially available consumables and equipment

**Fig. 6:**
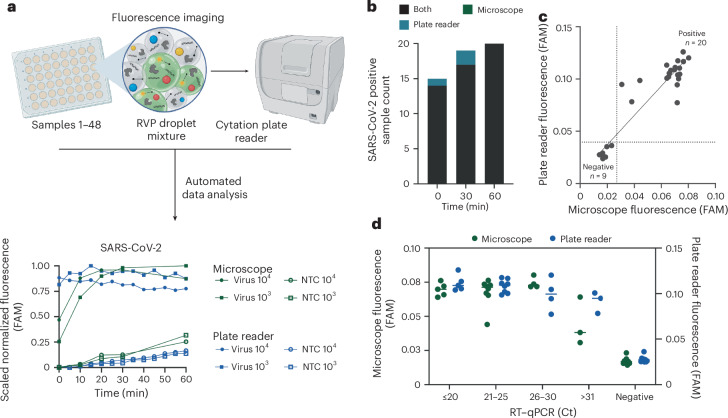
Automated readout of bbCARMEN reduces hands-on time and customized equipment requirements. **a**, Schematic of plate-reader platform, including comparison of bbCARMEN detection kinetics on the two readout platforms. **b**, bbCARMEN SARS-CoV-2-positive hit call at time 0, 30 and 60 min. Green, positive only by microscope; blue, positive only by plate reader; black, positive by both. **c**, Comparison of fluorescence signal using synthetic targets. Thresholds for positivity are shown as dotted lines. **d**, SARS-CoV-2 patient sample detection compared between readout platforms. Ct values computed as means of three technical replicates.

To further simplify deployment of this assay in low-resource settings, we reconfigured bead loading and imaging steps to use a standard well plate and a laboratory plate reader instead of our previous custom flow cells and fluorescence microscope setup (Fig. 6a). We found that 96-well plates loaded with a thin layer of droplets contained too many overlapping droplets of each colour code for statistical significance in the final diagnostic calls. However, loading onto 48-well plates enabled the droplets to be spread out over a larger area, enabling us to distinguish between different colour codes.

We compared concordance of assay results between the custom microscopy setup and the imaging plate reader and found 100% concordance between the two readout platforms across synthetic material and patient specimens (Fig. 6b–d and Supplementary Fig. 24). Moreover, the plate reader method showed earlier discrimination between target conditions and NTC than our previous microscopy approach, suggesting an improvement in both assay workflow and performance (Supplementary Fig. 24). As such, the changes to bead loading and imaging reduce equipment requirements, labour intensity and data analysis expertise while maintaining the sensitivity, specificity and low-resource setting applicability of bbCARMEN (Supplementary Note).

## Discussion

In this study, we employ bead-based approaches to achieve point-of-need and point-of-care uses in CRISPR diagnostics. These technologies increase sensitivity and deployability in resource-constrained settings.

Our luminescence bead-based approach, bbLuc, provides an attractive alternative to traditional fluorescence-based diagnostics, showing increased sensitivity in synthetic and clinical specimens. Critically, enhanced sensitivity also has upstream effects on assay adaptation to new or emerging pathogens by reducing the optimization time required to meet a target LOD. Furthermore, a luminescence assay reduces equipment requirements in conventional assays by removing the need for a light source for fluorescence excitation.

By using a multiplexed bead-based system for point-of-care diagnostics, bbCARMEN addresses the substantial equipment and expertise requirements of other multiplexed systems^16,17^. bbCARMEN maintains excellent multiplexing ability and sensitivity by using beads as an operationally simple, inexpensive modality to perform multiplexed reactions with high specificity as shown in our clinical sample testing. Implementation of our viral respiratory panel assay further demonstrates the ease of adaptability and its potential to dramatically increase deployability in resource-constrained settings. Together, these technologies offer a versatile toolkit for addressing diverse diagnostic needs, from highly sensitive single-target detection to comprehensive multiplex screening.

bbLuc and bbCARMEN provide a foundational framework for CRISPR-based diagnostics, offering a complementary platform for recent innovations. Future work could explore how SCOPE^29^, which enables rapid, ultrasensitive and field-deployable CRISPR/Cas13a-based detection, might be enhanced by incorporating bbLuc’s more sensitive luminescent reporter. In addition, the REVERSE system of ref. ^30^, which improves Cas13a activation kinetics, or the systems of refs. ^30,31^, or leverage amplification cascade systems such as CLEAR^32^ and PASSPORT^33^ improve reaction efficiency, sensitivity and speed. These approaches could help further optimize bbCARMEN’s workflow, ensuring robust performance across a broader range of targets while maintaining cost effectiveness in high-throughput settings.

Despite these advancements, we acknowledge certain limitations and challenges in current systems. While our bbLuc system offers considerable advantages in sensitivity and ease of readout, we also note that many molecular diagnostic technologies, including PCR and CRISPR-based diagnostics, typically require an extraction protocol to release nucleic acid from viruses and deactivate proteases. These steps require additional pre-processing steps and can present challenges in resource-limited settings. To address this, our lab has previously developed extraction-free methods such as HUDSON (Heating Unextracted Diagnostic Samples to Obliterate Nucleases) and demonstrated the effectiveness of commercial solutions such as the Intact Lysis Buffer. These methods have been successfully integrated with other CRISPR-based diagnostic platforms, and we believe they can be broadly applied in the future to simplify workflows in resource-limited settings.

While our systems represent substantial advancements in sensitivity and multiplexing capabilities for low-cost diagnostics, we recognize that the operational requirements for molecular diagnostics are generally more complex than those of antigen tests, which remain the gold standard for ease of deployment in resource-limited settings. However, CRISPR-based systems offer crucial advantages compared with both antigen testing and qPCR testing. They provide higher sensitivity than antigen testing, with SHINE demonstrating 50× improved sensitivity and bbLuc offering a further 20× boost compared with conventional fluorescent probes. Moreover, CRISPR-based systems such as bbLuc reduce equipment needs by using isothermal amplification instead of thermal cycling and by eliminating the need for fluorescence measurement. Our ongoing work aims to further simplify these requirements, potentially enabling room-temperature operation to make our technology more accessible across diverse settings.

Looking ahead, there are future directions that can further enhance these technologies. For example, in bbLuc, we focused on the cleavage of HiBiT nanoparticles instead of LgBiT nanoparticles. Due to manufacturing constraints, we could not consistently manufacture an RNA linker long enough for Cas13 cleavage of LgBiT nanoparticles, but future work may incorporate new technologies in RNA synthesis or protein-oligo conjugation. While refining bbLuc, we also considered sources of background signal inherent to dual bead systems. Although such systems can be susceptible to non-specific thermodynamic self-assembly, we found that careful optimization of conjugation chemistry, buffer composition and component concentrations resulted in a strong-enough cleavage signal to overcome background, enabling reliable detection and 100% specificity. These design choices helped ensure that Cas13-mediated cleavage produced a consistently distinguishable signal, even at low target input concentrations. We anticipate that future work may incorporate the use of cleavable on-bead LgBiT inhibitors (‘DrkBiT’) to further reduce background signal from HiBiT–LgBiT interactions^34^. In bbCARMEN, we were able to successfully resolve 9 different crRNA colour codes, giving resolution to discriminate against different viral infections in a point-of-care setting. However, future work can improve the number of simultaneously assayable viruses by iterating on colour code technology, either using new fluorescent dyes, a different combinatorial barcoding strategy, or multicolour bead approaches.

In conclusion, bbLuc and bbCARMEN, as independent advancements in single-plexed and multiplexed assays, respectively, highlight the potential of additional platforms to address diverse diagnostic needs, from bridging the gap between antigen tests and qPCR, to reducing the cost of multiplexed diagnostics. These technologies pave the way for diagnostic tools that can be tailored to specific applications and resource constraints. Ultimately, these platforms represent a step forward in CRISPR diagnostics, pushing the boundaries of sensitivity, portability and accessibility, and opening new avenues for the rapid and accurate detection of biological molecules in various settings.

## Methods

### Clinical samples and ethics statement

The use of excess human specimens, including nasopharyngeal swabs from Boca Biolistics, by the Broad Institute was reviewed and approved by the MIT Institutional Review Board (IRB) under protocol no. 1612793224. Human specimens from patients with SARS-CoV-2, HCoV-HKU1, HCoV-NL63, FLUAV, FLUBV, HRSV and HMPV were obtained under a waiver of consent from the Mass General Brigham IRB (protocol no. 2019P003305).

### Oligonucleotide sequence information

All nucleic acid sequences, including gBlocks, primers, crRNA, fluorescent oligos and linkers, are available for bbLuc and bbCARMEN in Supplementary Tables 1 and 2, respectively. All oligonucleotides used in this study were ordered from Integrated DNA Technologies (IDT). For ease of replicability, the sequences in the supplementary tables are provided in a format compatible with IDT’s ordering system.

### Sample collection and extraction

Patient samples were collected and stored in universal transport medium (UTM) or viral transport medium (VTM) and stored at −80 °C. Samples for luciferase reporter testing were extracted using automatic nucleic acid extraction on the KingFisher Flex Magnetic Particle Processor with 96 Deep Well Head (Thermo Fisher) using MagMAX mirVana Total RNA Isolation kit (Thermo Fisher, A27828) or MagMAX Prime Viral/Pathogen NA Isolation kit (Thermo Fisher, A58145), followed by an optional DNA cleanup using TURBO DNase (Thermo Fisher, AM2238). For bbLuc, RNA was extracted from 100 μl of input volume, eluted into a final volume of 16 μl water and stored at −80 °C. For bbCARMEN, RNA was extracted from 140 μl of input volume, eluted into a final volume of 50 μl water and stored at −80 °C.

### Bead preparation and coupling for bbLuc

HiBiT peptides were ordered from Promega as HaloTag protein conjugates (Promega, N3010), or custom-ordered through GenScript as a peptide with a leading glycine-serine linker (GSSGGSSG-VSGWRLFKKIS) with either an N-terminal azido-lysine modification (for SPAAC) or an N-terminal maleimide modification (for maleimide–thiol reactions). Biotinylated RNA linkers (Supplementary Table 1) were attached to the HiBiT peptides using either SPAAC or maleimide–thiol coupling. In some cases (Supplementary Figs. 7 and 8), PEG spacers (BroadPharm, BP-25731, BP-25730) were used with maleimide–thiol coupling to extend the length of the linkers. SPAAC coupling was done at room temperature overnight at a 3:1 ratio of azide-conjugated peptide to DBCO-conjugated oligo; maleimide–thiol coupling was done using a 20:1 ratio of maleimide-conjugated peptide to thiolated oligo left at room temperature for 2 h, with an additional overnight incubation at 4 °C.

For bead coupling, M270 Dynabeads were removed from stock and washed using magnetic separation three times with 1-min incubations in 1× binding and washing (BW) buffer with Tween (5 mM Tris-HCl pH 7.5, 0.5 mM EDTA, 1 M NaCl, 0.0125% Tween-20). After washing, beads were resuspended in twice the volume of 2× Wash Buffer with Tween (10 mM Tris-HCl pH 7.5, 1 mM EDTA, 2 M NaCl, 0.025% Tween-20). Beads were then combined in 1× PBS with HaloTag-LgBiT protein (Promega, CS1967B01) and HaloLigand-Biotin (Promega, G8281) at a 1:1.5 stoichiometric ratio (with HaloTag-LgBiT protein concentration of 80 nM unless otherwise noted). Following a 30-min incubation period on a rotator and another round of bead washing as above, LgBiT beads were resuspended in Tween buffer (1,000 μl PBS/0.05% Tween-20/0.1% BSA) and HiBiT beads were resuspended in 1× TEL buffer (0.0125% Tween: 5 mM Tris-HCl pH 7.5, 0.5 mM EDTA, 10 mM NaCl, 0.0125% Tween-20), followed by storage at 4 °C.

### Fluorescence and luciferase-based Cas13 assays for bbLuc development

Fluorescence-based reactions were conducted as previously described in refs. ^6,14–18^ as a point of comparison for luminescence-based reactions and used a polyU FAM reporter. RPA primers and crRNa were designed and selected as described previously^6,14–18^.

For amplification-free assays, *Leptotrichia wadei* Cas13a (*Lwa*Cas13a) protein was first resuspended to 465.7 nM in 1× SB medium (50 mM Tris-HCl pH 7.5, 600 mM NaCl, 2 mM DTT, 5% glycerol). A master mix was created with the following reagents: 1× CB buffer (40 mM Tris-HCl pH 7.5, 1 mM DTT**)**, 22.5 nM crRNA, 2 U μl^−1^ RNase Inhibitor Murine (NEB, M0314), 46.6 nM *Lwa*Cas13a (GenScript) and 40 mM polyU FAM or 20 μg μl^−1^ LgBiT and 20 μg μl^−1^ HiBiT beads. In luciferase reactions, beads were washed in 1× TEL buffer (5 mM Tris-HCl pH 7.5, 0.5 mM EDTA, 10 mM NaCl, 0.0125% Tween) using a process similar to that outlined above, before being added to the final mix. While the final reaction concentrations of bead-bound LgBiT and HiBiT were 80 nM and 300 nM, respectively, these concentrations were varied in experiments as described above to identify optimal conditions. Fluorescence or luminescence kinetics were measured at 37 °C on a Biotek Cytation 5 plate reader using a 384-well low-flange white flat-bottom polystyrene NBS microplate (3574) or Corning 384-well microplate (3821), respectively.

For amplification reactions (SHINE), the reaction was done as previously shown in refs. ^6,14–18^. *Lwa*Cas13a protein was first resuspended to 2,250 nM in 1× SB (50 mM Tris-HCl pH 7.5, 600 mM NaCl, 2 mM DTT, 5% glycerol). The master mix was created in 1× SHINE buffer (20 mM HEPES pH 8.0, 60 mM KCl, 5% PEG-8000) and included 45 nM *Lwa*Cas13a protein, 1 U μl^−1^ RNase Inhibitor Murine (NEB, M0314), 2 mM of each rNTP (NEB, N0450), 1 U μl^−1^ NextGen T7 RNA polymerase, 2 U μl^−1^ Invitrogen SuperScript IV (SSIV) reverse transcriptase (Thermo Fisher, 18090010), 0.1 U μl^−1^ RNase H (NEB, M0297S), 14 nM magnesium acetate (Millipore Sigma, 63052), 140 nM RPA primers for bbLucV0 and 60 mM primers for bbLucV1 (and comparator fluorescence assays), 22.5 nM crRNA, and for fluorescence SHINE, 40 nM polyU FAM reporter. The reaction was created in reaction units of 107.5 μl, with one RPA pellet (TwistDx, TABAS03KIT) per reaction unit.

In the case of luciferase reactions, beads were washed as above in 1× TEL buffer, then resuspended to a 4× concentration in 5 mM HEPES buffer pH 8.0. Final reaction concentration was 5 µg µl^−1^ beads each for HiBiT and LgBiT beads. Furimazine was added as described, with a final concentration of 50 µM in bbLucV0 and 150 µM in bbLucV1 SHINE. Critically, in bbLucV0 we added furimazine to the 4× bead resuspension before addition to the master mix, but in bbLucV1, we added the furimazine directly to the master mix to prevent salting out at the higher furimazine concentration. Finally, 5% by volume target or sample was added to the reaction before measurement for 3 h at 37 °C in a Biotek Cytation 5 for continuous readouts. For single-timepoint readouts in Nigeria and where described, the Luminescence 96 by Byonoy was used as an imaging modality, with a standard 37 °C incubator or a thermal cycler set at 37 °C (to simulate an isothermal heat block) used for reaction incubation, and reactions incubated in low-volume white 96-well plates (Revvity, 66PL96025). Fluorescence data were collected in Nigeria using the BioRad CFX96 machine, with fluorescence values background subtracted using the first timepoint for each well and overall baseline adjusted to the lowest collected fluorescence value, as recommended by the manufacturer. For smartphone-based detection, the same overall reactions were used as described above for bbLuc, scaled up to 100 μl. Reactions were conducted in a thermal cycler set at 37 °C (to simulate an isothermal heat block) and imaged in a dark room using the default camera application in an iPhone 14.

Iterative optimization of the reaction was done via modification of reagent and bead concentrations as described in each experiment. Optimal conditions that produced the lowest limit of detection were incorporated into the final protocol as described. In each optimization experiment, the reaction component that was changed is outlined in the results or figures. The following conditions remained constant across experiments: 45 nM *Lwa*Cas13a protein resuspended in 1× SB (such that resuspended protein is at 2.26 μM), 1 U μl^−1^ murine RNase inhibitor and 2 mM of each rNTP.

For fluorescence data shown using kinetic timepoint-based readouts, fluorescence values were normalized across condition by dividing timepoint data by the mean NTC signal at the first-collection timepoint. Signal was deemed positive if a replicate signal was over 3 standard deviations away from the mean NTC. For luminescence data for amplification-free conditions (including curves) and timepoint curves for SHINE, luminescence values were normalized across condition by dividing timepoint data by the mean NTC signal at the first-collection timepoint. For luminescence SHINE data, the larger kinetic complexity precluded the use of a single timepoint to determine a positive/negative call. As such, calls are shown as luminescence ratios, and an overall measure of signal across the timepoint curve was determined. This was done by first aligning experimental and NTC condition slopes (as computed between the timepoint nearest to 12 min and its subsequent timepoint) by dividing experimental condition by the NTC slope, and next by finding the ratio of the sum of intensities across the experimental and NTC conditions. Patient samples were determined positive with a signal threshold >1.35. For both luminescence SHINE and fluorescence SHINE data compared at single timepoints, signal was normalized to the average NTC of the timepoint; and in luminescence SHINE, a signal was determined as positive if it exceeded 1.35× the average NTC and 3× the standard deviation of the NTCs at that timepoint.

### Bead preparation and coupling for bbCARMEN

Streptavidin-coated polystyrene beads (Spherotech, SVP-200-4) were washed and stored in 2× BW buffer (10 mM Tris-HCl pH 7.5, 1 mM EDTA and 2 M NaCl). To prepare beads for BSA coating, 1 ml of beads was washed with 1 ml of 1× BW buffer three times before being resuspended in 2 ml of 2× buffer and 2 ml BSA (4 mg ml^−1^) (NEB, B9000). Beads were BSA blocked for 3 h on a rotating stand at room temperature before washing with 1× BW buffer twice, and resuspended in twice the original volume of 2× BW buffer. BSA blocked beads were stored at 4 °C until use at a 2.5 μg μl^−1^ bead concentration.

crRNA and dye coupling were split into two separate steps. First, 32 nM of desired crRNA was mixed with BSA blocked beads at a 1:1 ratio and incubated at room temperature for 15 min. After the coupling incubation, crRNA beads were washed with 1× BW buffer once before resuspending in the original volume of beads with 2× BW buffer. Equal volumes of pre-mixed colour-coding dyes were added to the corresponding crRNA bead and incubated at room temperature for 15 min. The ratios of AF546, AF594 and AF647 in each corresponding target were as follows: SARS-CoV-2, 50:0:0; HCoV-HKU1, 48.5:0:1.5; BetaCov, 37.5:0:12.5; Scrambled1, 0:50:0; FLUBV, 30:20:0; HRSV, 47.5:2.5:0; RNase P, 0:0:50; HCoV_NL63, 0:37.5:12.5; HPIV3, 0:45:5; FluA, 0:48.5:1.5; HMPV, 45:3.5:1.5; and Scrambled2, 35:13.5:1.5. Colour-coded crRNA beads were washed six times with 1× BW buffer and then resuspended in 1× TEL buffer (5 mM Tris-HCl pH 7.5, 0.5 mM EDTA, 10 mM NaCl) to have a final bead concentration of 2.5 μg μl^−1^. crRNA beads were stored at 4 °C until use and washed twice before pooling for each experiment. Equal volumes of beads were pooled together the day of an experiment and incubated in a bbCARMEN wash buffer (Cas13 detection master mix without Cas13, 10× cleavage buffer and viral target) for 60 min and washed twice with 1× BW buffer. All washes were accomplished by spinning at 15,000 r.c.f. on a tabletop centrifuge and discarding the supernatant. Non-BSA blocked beads required spin times of 3.5 min, while BSA blocked beads required 1.5 min.

### Flow cell design and fabrication for bbCARMEN

Flow cell dimensions were designed in AutoCAD (AutoDesk) and optimized by empirical testing to increase sample size and loading speed. To be compatible with existing imaging instruments, the size of a standard microscope slide (25 × 75 mm) was selected. The optimal lane geometry was achieved by maximizing the number of droplets captured in a single-lane image field of view. To allow for easy loading, eight 10.5 × 5.8 mm lanes were spaced out on the 75-mm-long flow cell, with inlet spacing of 9 mm for compatibility with 8-channel multichannel pipettes. Standard-size flow cells contain two rows of 8, for 16 samples per device. Increasing flow cell dimensions to 50 × 75 mm enabled 32-lane imaging per device.

All flow cells were fabricated with acrylic, a single layer of double-sided clear film tape and hydrophobic treated glass slides. In brief, 12 inch × 12 inch cast acrylic sheets (1/4 inch or 1/8 inch, clear) were purchased from Amazon (Small Parts, B004N1JLI4) and were cut using an Epilog Fusion M2 laser cutter (60 W), producing an acrylic cover with inlets and outlets. Sheets of clear film tape were cut on the laser cutter to provide the geometry of the lanes. Untreated glass slides were treated with Aquapel from Amazon (Aquapel, 2PACK_A) to create a hydrophobic surface. For assembly of both the 16- and 32-lane flow cells, the clear tape was first adhered to the Aquapel-treated glass slide and then to the acrylic cover. Flow cells were stored in plastic bags at room temperature until use.

### Single-step amplification for bbCARMEN

All targets for RVP2.0 were amplified in a multiplexed PCR reaction using the QIAGEN OneStep RT–PCR Mix. A total reaction volume of 50 μl was used with some modifications to manufacturer-recommended reagent volumes, specifically a 1.25× final concentration of OneStep RT–PCR buffer, 2× more QIAGEN enzyme mix and 20% RNA input. For optimal amplification, final viral primer concentrations varied, with SARS-CoV-2, HCoV_NL63, HCoV_OC43, HPIV3 and HMPV primer concentrations at 300 nM, HCoV_HKU1 and HRSV at 600 nM, FluA and FluB at 480 nM, and RNase P at 100 nM. The following thermal cycling conditions were used: (1) reverse transcription at 50 °C for 30 min; (2) initial PCR activation at 95 °C for 15 min; and (3) 40 cycles at 94 °C for 30 s, 56 °C for 30 s and 72 °C for 30 s.

### Cas13 detection in bbCARMEN

Detection assays were performed with 45 nM purified *Lwa*Cas13a, 0.5 μg μl^−1^ of pooled crRNA beads, 500 nM quenched fluorescent RNA reporter, 1 μl murine 40,000 U ml^−1^ RNase inhibitor (New England Biolabs) in nuclease assay buffer (40 mM Tris-HCl, 60 mM NaCl, pH 7.3) with 1 mM ATP, 1 mM GTP, 1 mM UTP, 1 mM CTP and 0.6 μl T7 polymerase mix (Lucigen).

### Emulsification, loading and imaging in bbCARMEN flow cells

For emulsification, detection samples (10 μl) were mixed with 2% 008-fluorosurfactant (RAN Biotechnologies) in fluorous oil (3M 7500, 35 μl) in a 96-well plate. Plates were sealed and physically shaken vertically for up to 30 s and then spun down for 15 s.

For loading, 30 μl of excess oil was removed from each emulsion before 9 μl of droplets were loaded into a 16- or 32-lane bbCARMEN flow cell. The background negative control was computed by analysing fluorescence signals from droplets containing a scrambled crRNA sequence attached to a colour-coded bead. Flow cells were sealed with a PCR film to prevent evaporation of samples.

All bbCARMEN flow cells were imaged on a Nikon TI2 microscope equipped with an automated stage (Ludl Electronics, Bio Precision 3 LM), LED light source (Lumencor, Sola) and camera (Hamamatsu, Orca Flash4.0, C11440, sCMOS) using a ×2 objective (Nikon, MRD00025). The following filter cubes were used for imaging: Alexa Fluor 405: Semrock, LED-DAPI-A-000; Alexa Fluor 555: Semrock, SpGold-B; Alexa Fluor 594: Semrock, 3FF03-575/25-25 + FF01-615/24-25; and Alexa Fluor 647: Semrock, LF635-B. During imaging, the microscope condenser was tilted back to reduce background fluorescence in the 488 channel. Unless otherwise specified, all flow cells were imaged three times over the course of 60 min, with an incubation at 37 °C between T30 and T60.

### SCoV2 RT–qPCR protocol

To detect the presence of SARS-CoV-2 RNA, the extracted RNA samples underwent testing using CDC’s SARS-CoV-2 RT–qPCR assay (2019-nCoV CDC EUA kit, IDT, *Taq*Path 1-Step RT–qPCR Master Mix, CG) targeting the N1 and RP regions. The cycling conditions for the RT–qPCR were as follows: an initial hold at 25 °C for 2 min, followed by reverse transcription at 50 °C for 15 min, polymerase activation at 95 °C for 2 min, 45 cycles of denaturation at 95 °C for 3 s, and annealing/elongation at 55 °C for 30 s. RT–qPCR analysis was performed using a QuantStudio 6 instrument from Applied Biosystems, and the data were analysed using the Standard Curve module of the Applied Biosystems analysis software.

### Automated bbCARMEN data analysis

CellProfiler^35^ and a custom Jupyter notebook were used to automate image analysis for bbCARMEN^36^. First, beads were identified and measured in red, yellow, green and blue channels using a CellProfiler pipeline. Briefly, images were illumination corrected by subtracting an approximation of image background from each channel. Then, bleedthrough between colour channels was computationally compensated for by image subtraction. The corrected images were then masked to exclude the edges of the wells where droplets piled up. Beads were filtered by shape (solidity, eccentricity) to exclude debris and by number of neighbours to exclude beads that were very close to other beads. Beads were also associated with droplets and excluded if a droplet contained multiple beads. The object mask for each accepted bead was expanded 5 pixels and the original bead area subtracted from this to form a ‘donut’ shape in which intensity in the droplet blue channel was measured. For each bead, we calculated normalized intensity measurements for each (red, yellow, green) channel by dividing the mean intensity measurement for each channel by the sum of the mean intensities across all 3 channels. Finally, beads were also tracked across images taken at different timepoints using the linear assignment problem (LAP) framework^37^.

CellProfiler measurements were used for bead classification and FAM fluorescence measurement in a separate downstream analysis Jupyter notebook. Beads were clustered using normalized red, green and yellow intensity measurements for each bead using *k*-means clustering. Results are displayed in a ternary plot showing each bead’s intensity in green, yellow and red channels, and beads were coloured by cluster (Supplementary Fig. 14). Each cluster was then associated with a virus from the panel by measuring the distance from its measured centroid to the default centroid locations (provided by an external file) and selecting the label of the closest default cluster centroid. In this process, additional QC plots and metrics were also generated and used to assess data quality. Finally, bead donut median blue channel intensities were used to classify samples as positive or negative for each virus in the virus panel, using a threshold based on either fold difference from negative control beads (in the same well) and/or exceeding a number of standard deviations above the median intensity of negative control beads. Tracked bead blue channel fluorescence was also plotted over time to observe kinetics of FAM fluorescence. The code for this pipeline is available in GitHub at https://github.com/broadinstitute/bbCARMEN_analysis/ (ref. ^38^).

### Reporting summary

Further information on research design is available in the Nature Portfolio Reporting Summary linked to this article.

## Supplementary information

Supplementary Information Supplementary Note, Figs. 1–24 and Tables 1–3.

Reporting Summary

## Data Availability

The data, code and detailed methods used in the design of primers and crRNAs are available at adapt.sabetilab.org. Any other relevant data are available from the authors on reasonable request.

## Code availability

The code for the analysis of bbCARMEN data is available in GitHub at https://github.com/broadinstitute/bbCARMEN_analysis/ (ref. ^38^).
