# Supplementary material for: Bead-based approaches for increased sensitivity and multiplexing of CRISPR diagnostics
Source: Nat Biomed Eng. 2025 Sep 22;10(5):939–51. doi: 10.1038/s41551-025-01498-2 (PMC13190306; doi:10.1038/s41551-025-01498-2)
Supplement: Supplementary file 1 — Supplementary Note, Figs. 1–24 and Tables 1–3. [file 41551_2025_1498_MOESM1_ESM.pdf]

nature biomedical engineering

Supplementary information

https://doi.org/10.1038/s41551-025-01498-2

Bead-based approaches for increased sensitivity and multiplexing of CRISPR diagnostics

In the format provided by the authors and unedited

Supplementary information

Supplementary Note - Page 2

Supplementary Figures - Page 4

Supplementary Tables - Page 28

Supplementary Note: Operational and Economic Advantages of bbCARMEN

bbCARMEN introduces a more accessible and scalable approach to multiplexed CRISPR-based diagnostics by reducing both capital investment and ongoing operational costs. Below, we outline how these improvements manifest across different testing scenarios and resource settings.

Reduced Capital Costs

bbCARMEN eliminates the need for multiple qPCR machines by consolidating all targets into a single reaction per sample. Traditional high-throughput qPCR workflows require a substantial number of machines to achieve multiplexed testing at clinical scale. For example, testing 96 samples for 10 targets would demand **960 wells per run**. Even under optimistic assumptions—such as 4-target multiplexing with single replicates—this setup still requires **4 qPCR machines** to accommodate throughput. In more common **non-multiplexed workflows**, 10 qPCR machines would be required.

By contrast, bbCARMEN compresses all 10 targets into a single dropletized reaction per sample, which can be performed on a standard 96-well plate imager without the need for microfluidics or target-specific qPCR modules. This architectural simplification reduces instrument requirements by 4–10× depending on the workflow and eliminates the need for proprietary hardware such as expensive microfluidic chips used in earlier CARMEN versions.

Simplified Workflow and Reduced Labor Burden

By replacing dozens of reactions with one assay, bbCARMEN streamlines diagnostics and minimizes hands-on time. bbCARMEN consolidates what would traditionally require dozens of separate reactions into a single streamlined workflow, minimizing hands-on time, complexity, and training requirements. In qPCR-based workflows, multiplexed diagnostics involve numerous pipetting steps, reaction setups, and machine programming across multiple thermal cyclers. Maintaining replicates, preventing cross-contamination, and tracking reaction layouts all increase the labor burden.

bbCARMEN streamlines this entire process through:

- Equipment-free droplet generation, enabling low-cost reaction multiplexing
- A single plate-based readout with automated filter switching
- A standardized data analysis pipeline

These features dramatically reduce operational complexity. As a result, bbCARMEN is easily deployable in both centralized and decentralized settings without sacrificing quality or consistency.

Lower Per-Sample Costs Across Scales

bbCARMEN reduces per-sample reagent use by allowing one to test for all targets in a single

reaction, enabling cost savings at both small and large scales. In qPCR-based systems, per-sample costs are proportional to both the number of reactions and the number of targets. A 10-target panel might require 10 to 30 reactions per sample depending on the number of replicates and multiplexing capacity, leading to steep reagent use and cumulative costs. Even under 4-target multiplexing, multiple wells are still needed per sample, driving up enzyme and probe consumption.

bbCARMEN miniaturizes detection by splitting each sample into ~175–225 parallel dropletized CRISPR reactions, drastically reducing reagent use and simplifying setup.These efficiency gains scale with testing volume, making bbCARMEN cost-effective at both low and high sample throughput. Importantly, unlike earlier CARMEN implementations that required custom microfluidic chips (costing >$500 each), bbCARMEN uses standard 96-well plates, keeping consumable costs low and sourcing flexible.

Scalability Across Resource Settings

While mCARMEN enables very high-throughput testing, bbCARMEN is better suited for cost-sensitive and flexible deployment. mCARMEN relies on custom microfluidic chips that cost >$500 each, making it ideal for large-scale centralized testing but less accessible in low-resource or lower-volume settings. In contrast, bbCARMEN uses standard 96-well plates, keeping per-run costs low regardless of batch size.

This makes bbCARMEN particularly well-suited for routine surveillance, mobile labs, and outbreak response, where testing volumes can vary from day-to-day and cost flexibility is essential.

Supplementary Figures

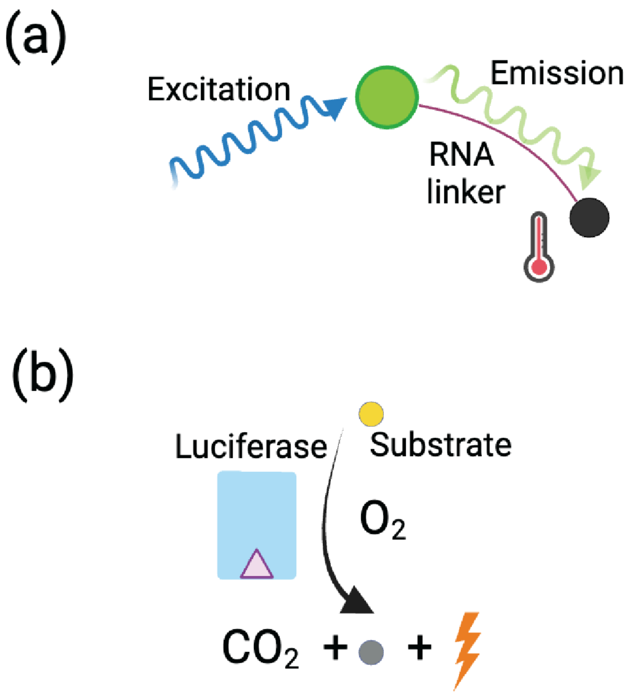

Supplementary Figure 1: Schematic of (a) quenched fluorescence vs (b) split luciferase-based reporter systems.

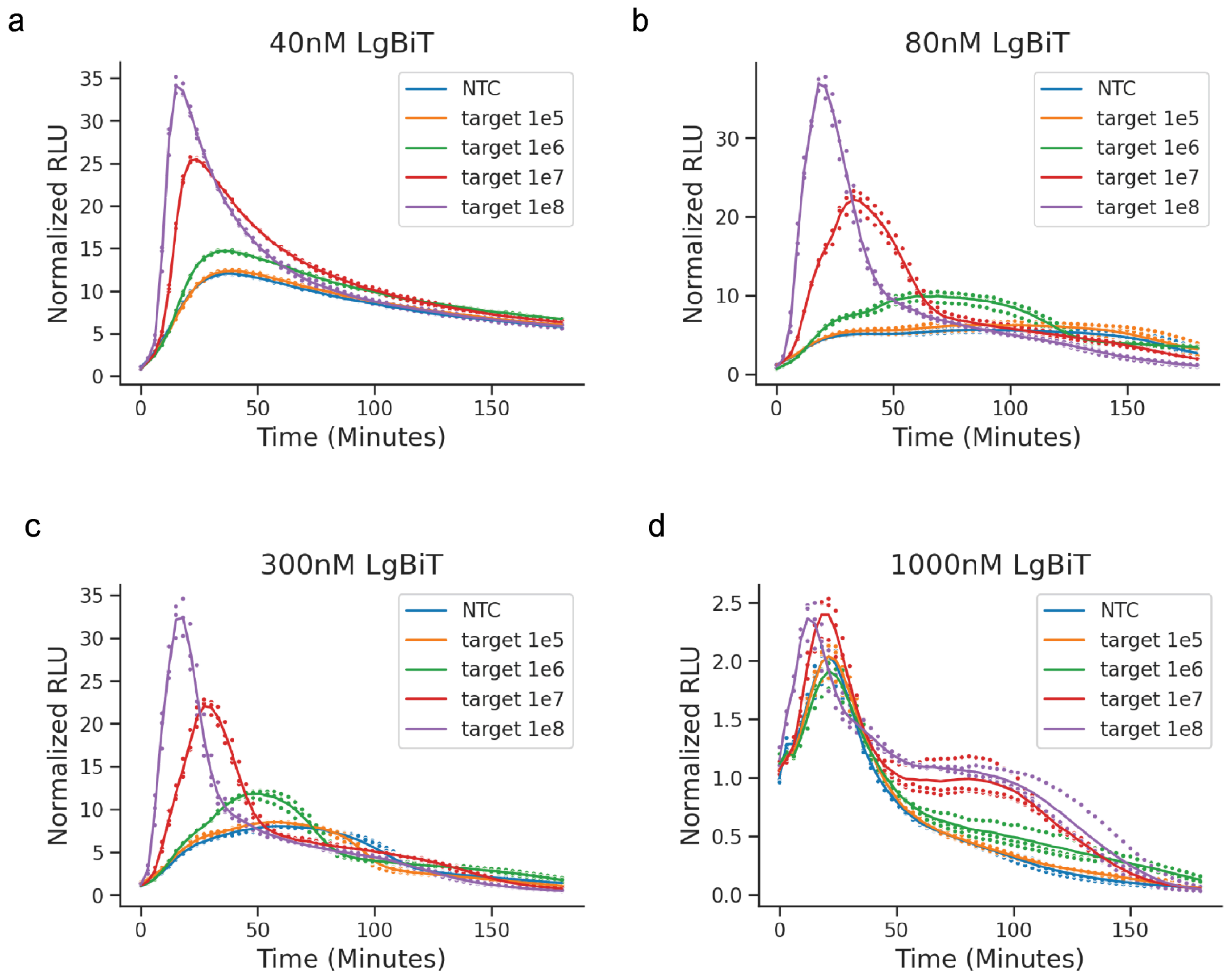

Supplementary Figure 2: Optimizing LgBiT Concentration. Detection-only luminescent reaction with different concentrations of LgBiT (40 nM, 80 nM, 300 nM, and 1000 nM) and 300 nM HiBiT after 3h on varied synthetic RNA target; NTC, no target control. 80nM and 40nM LgBiT concentrations showed similar detection efficiencies, with 1000nM LgBiT-np showing heavily reduced detection, likely due to saturation of LgBiT-np and HiBiT-np bead-bead interactions. 80nM LgBiT was ultimately chosen as it demonstrated a higher SNR of any of the conditions tested.

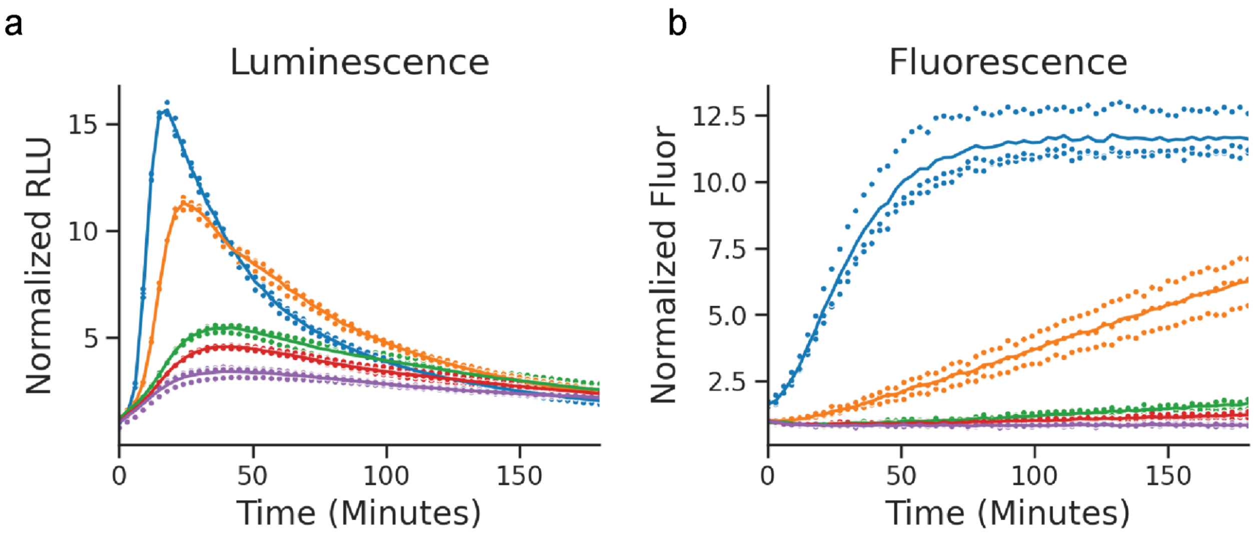

Supplementary Figure 3: Optimized luminescent amplification-free assay kinetics compared to fluorescent assay on varied synthetic RNA target, 3 hours (80nM LgBiT, 300nM HiBiT, 200uM furimazine).

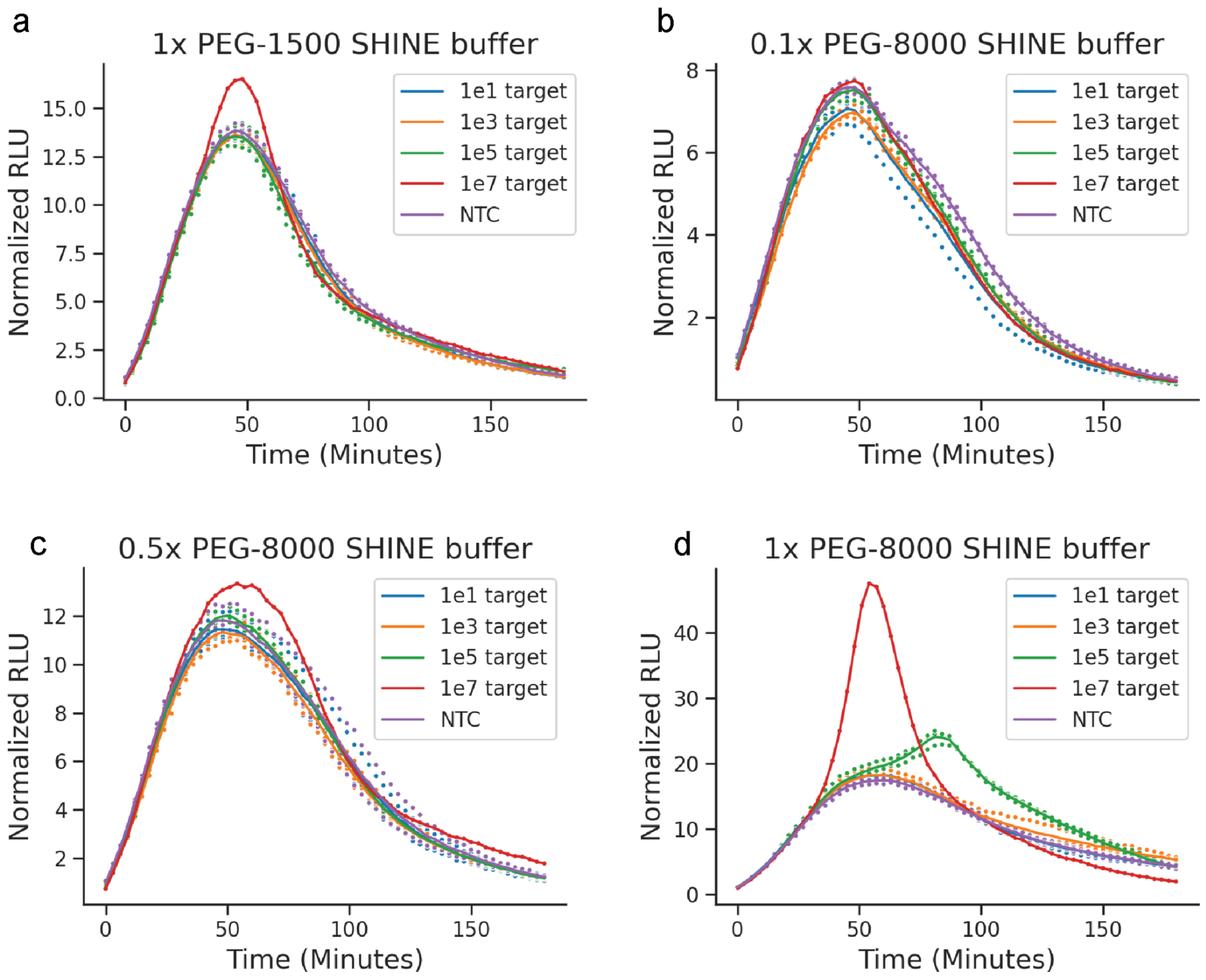

Supplementary Figure 4: Optimizing PEG Buffer Concentration. Luminescent SHINE was performed with varying makeup of PEG buffer, specifically 5% PEG-8000, 2.5% PEG-8000, 0.5% PEG-8000, and 5% PEG-1500 on synthetic RNA target; NTC, no target control. Optimal performance was observed with 5% PEG-8000, with other concentrations severely limiting performance.

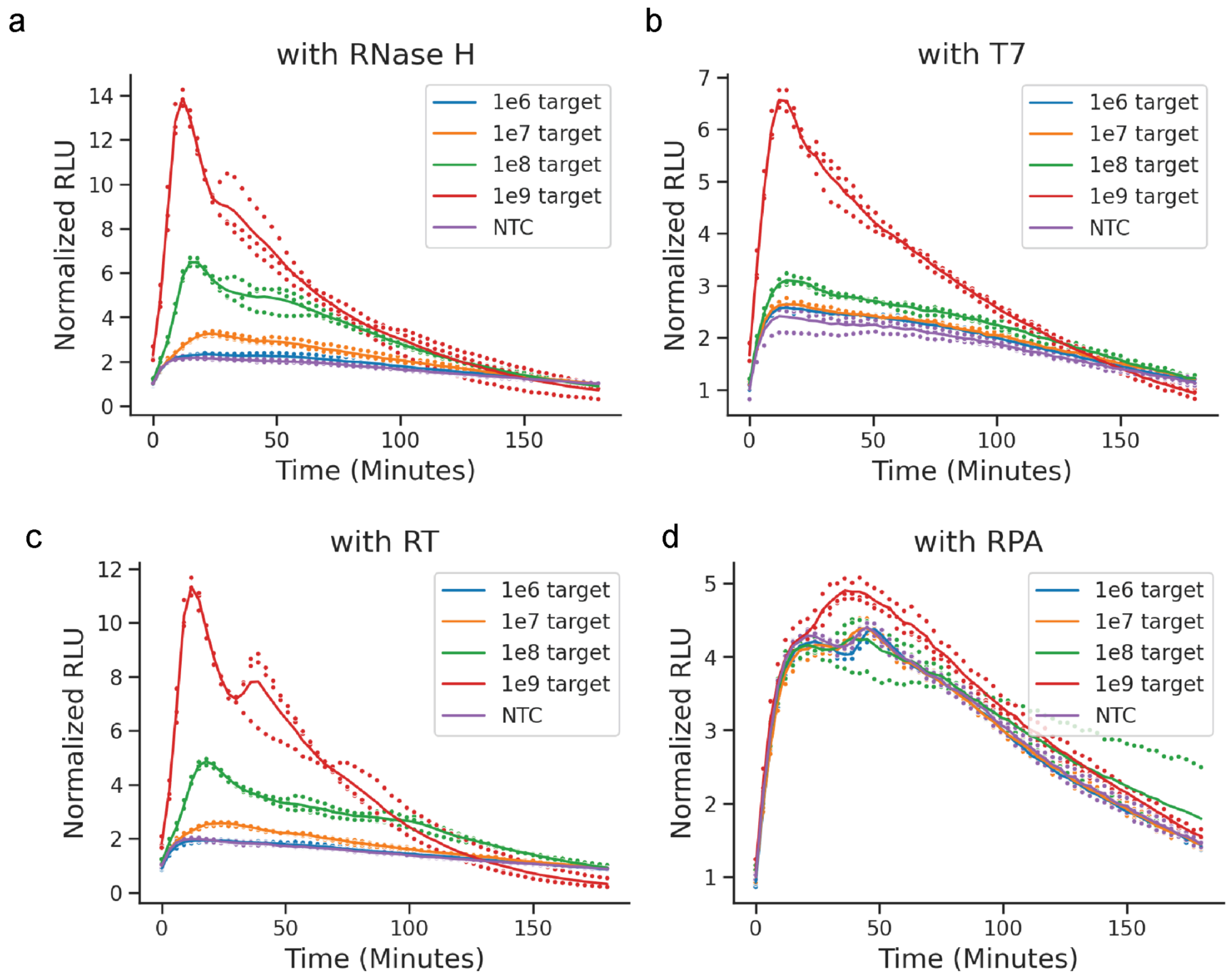

Supplementary Figure 5: Components of RPA pellets interfere with detection. Amplification-free reactions in SHINE buffer, with RPA pellets, RNase H, reverse transcriptase, and T7 RNA polymerase spiked in separately in different conditions. RNase H, reverse transcriptase, T7 RNA polymerase additions show little relative inhibition to detection in SHINE buffer. In contrast, addition of RPA pellets significantly degrades detection-only performance. Experiments done on varied synthetic RNA target; NTC, no target control.

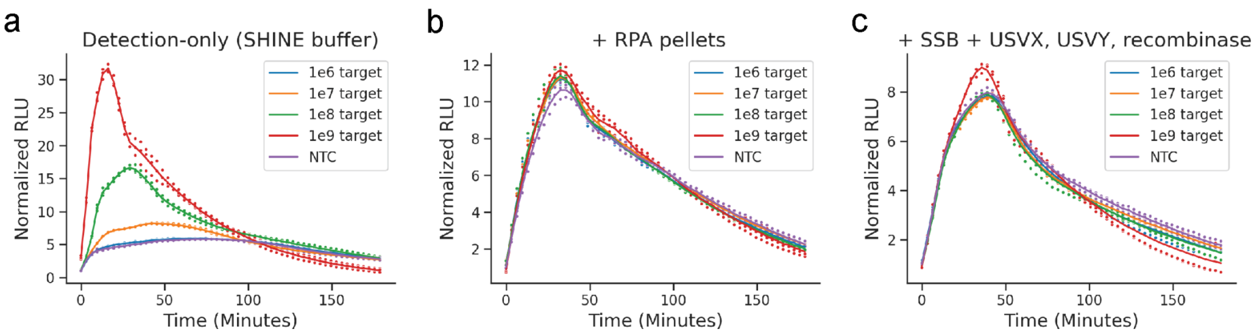

Supplementary Figure 6: RPA pellet components (SSB and recombinases) interfere in detection. Luminescent amplification-free assays, with optimized CB buffer substituted with SHINE buffer. Differing conditions with RPA pellets spiked in, and with constituent enzymes of RPA (SSB, USVX, USVY) added in RPA concentrations.

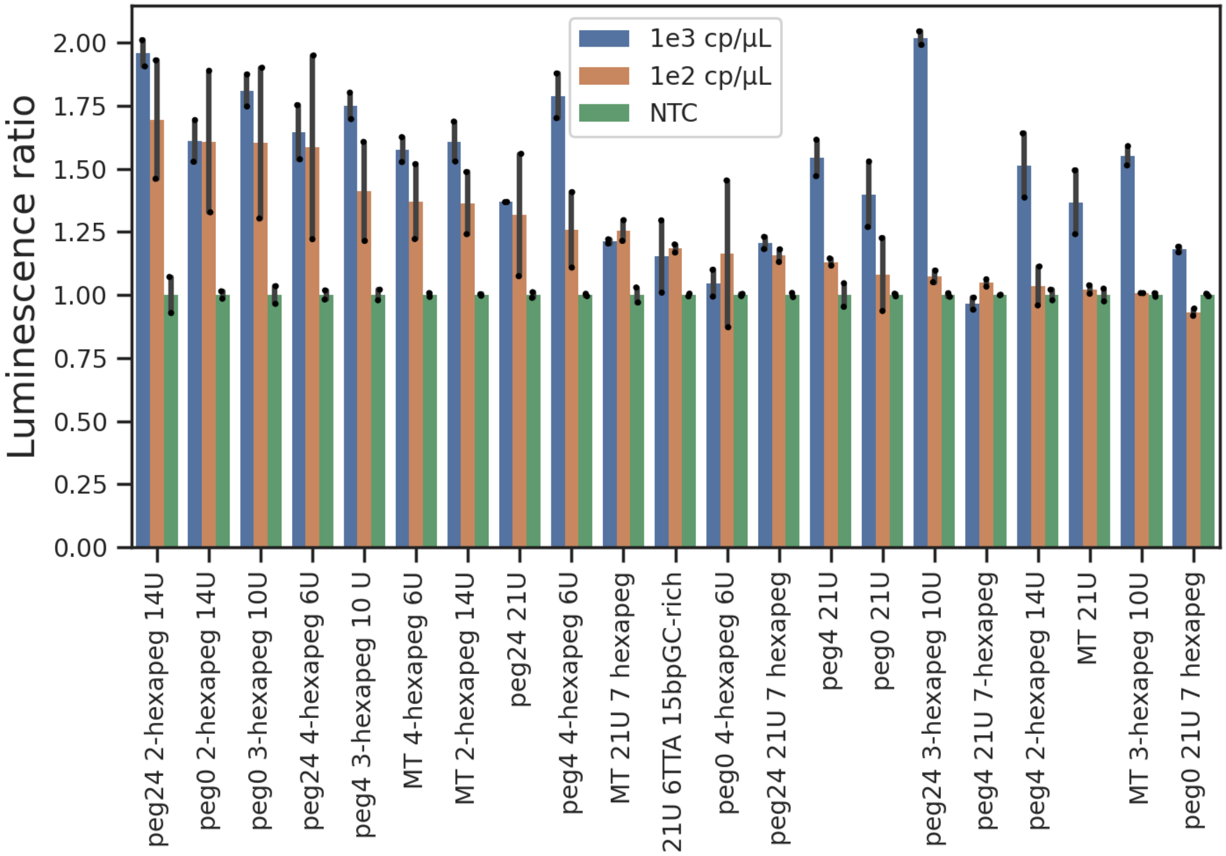

Supplementary Figure 7: Detection using 21 different RNA linkers**.** We tested several different designs of oligos with different types of conjugation (SPAAC, MT, DBCO-PEG-MT) and 8 types of oligos. Five different base oligos were used (Maleimide-thiol 21U Thiol, Thiol 21U 7-hexapeg, 2-hexapeg 14U, 3-hexapeg 10U, 4-hexapeg 6U). Three different conjugation methods were also used (Maleimide-Thiol and Maleiemide-Thiol-PEG-DBCO). We found that the following had the best detection. The bars are ordered as descending ratios of 10^2 copies/μL target to NTC signal. Error bars denote 95% confidence intervals.

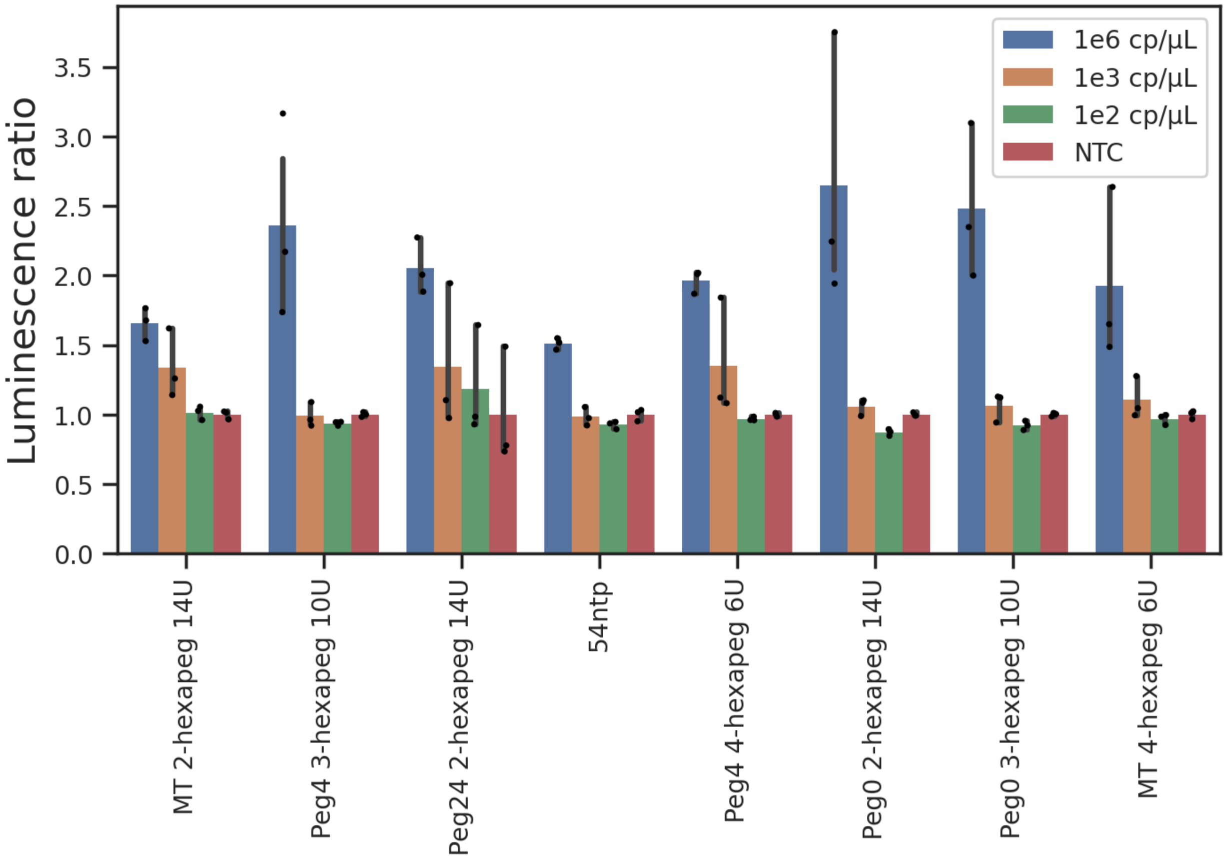

Supplementary Figure 8: Detection using 8 best RNA linkers. We tested several different designs of oligos with different types of conjugation (SPAAC, MT, DBCO-PEG-MT) and 8 types of oligos. The bars are ordered as descending ratios of 10^2 copies/µL target to NTC signal. Peg0 oligos refer to oligos where DBCO-PEGX-maleimide moiety attached to relevant linkers have X = 0, i.e. no peg. Error bars denote 95% confidence intervals.

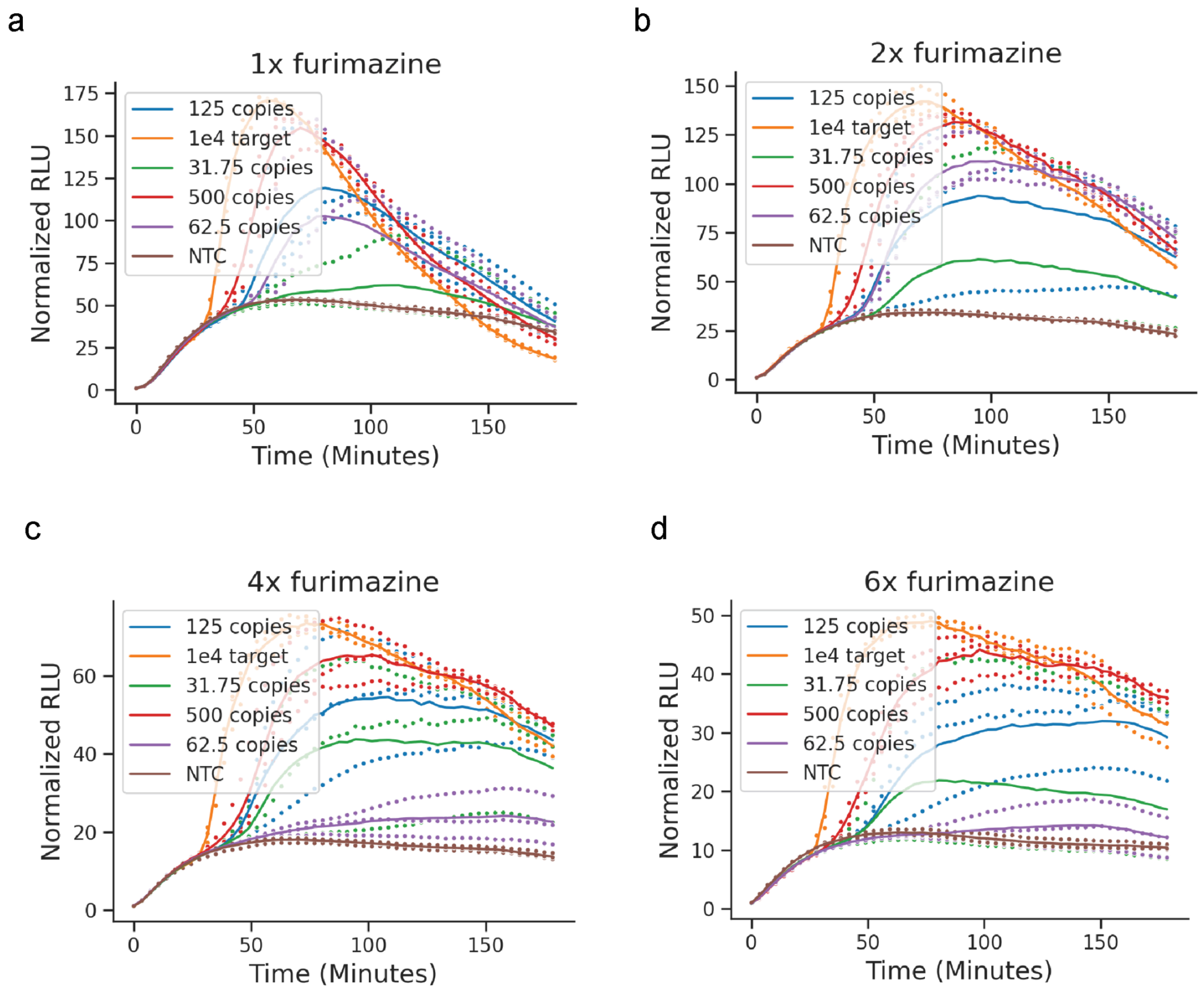

Supplementary Figure 9: Optimizing furimazine concentration. Luminescent SHINE assay showing different concentrations of furimazine (1x, 2x, 4x, 6x). 1X is 26μM furimazine

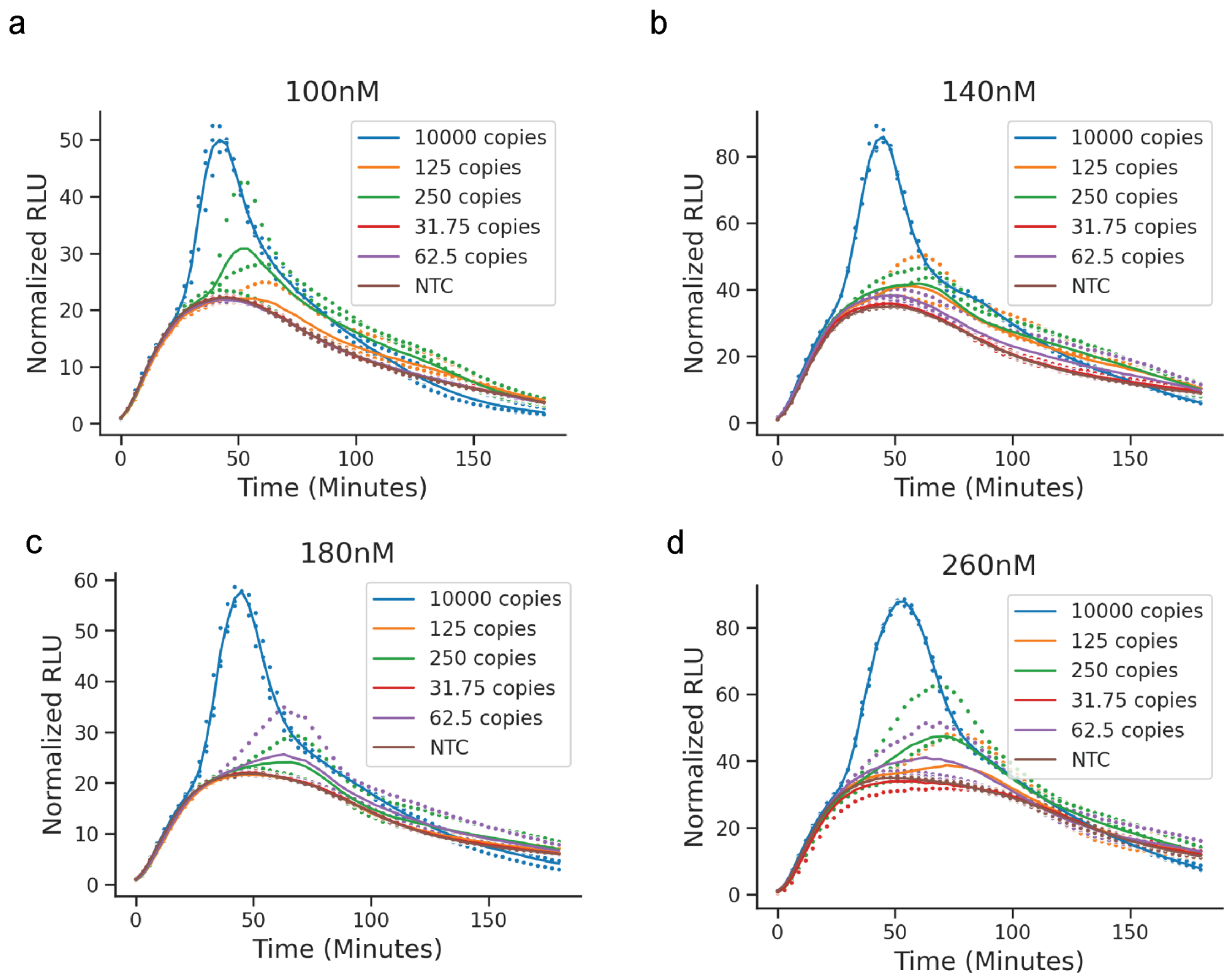

Supplementary Figure 10: Optimizing RPA primer concentration. Luminescent SHINE assay showing different concentrations of RPA primers (100 nM, 140 nM, 180 nM, 260 nM).

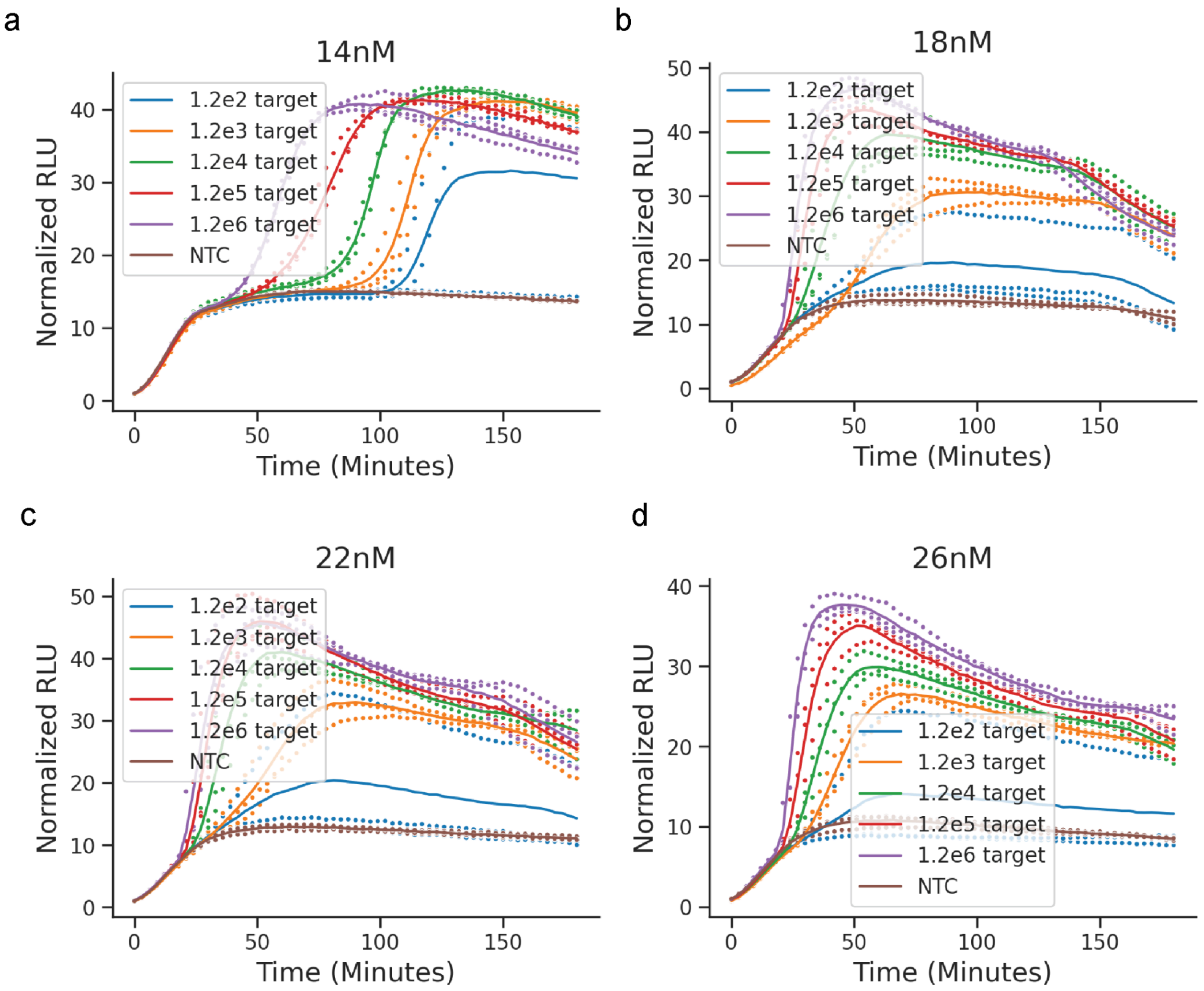

Supplementary Figure 11: Optimizing magnesium acetate concentration. We tested different concentrations of magnesium acetate. We found that 14 nM of MgOAc has the best signal-to-noise ratio at low sensitivity, however 18-26 nM had better speed (faster detection prior to 40 minutes).

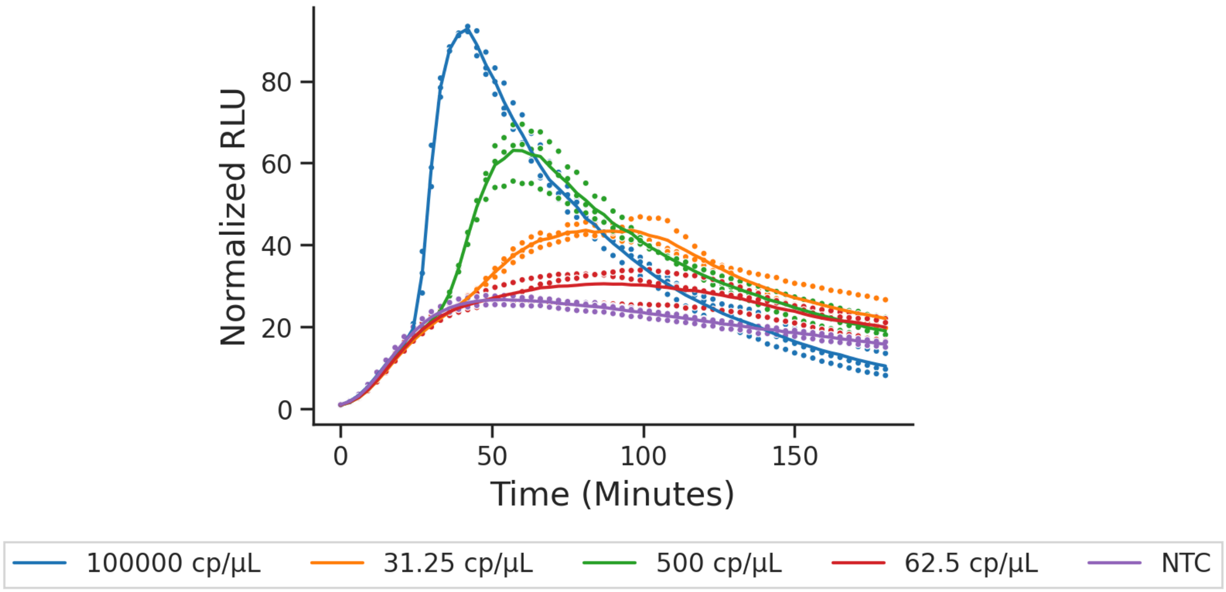

Supplementary Figure 12: limit of detection of bbLucV0 SHINE. Luminescent SHINE assay results for a dilution series of synthetic SARS-CoV-2 RNA, ranging from 10^6 to 31.25 copies/μL, using concentrations of furimazine (50 μM), RPA primers (140 nM), and magnesium acetate (14 nM). The assay demonstrates an analytical sensitivity of 32 copies/μL within 75 minutes.

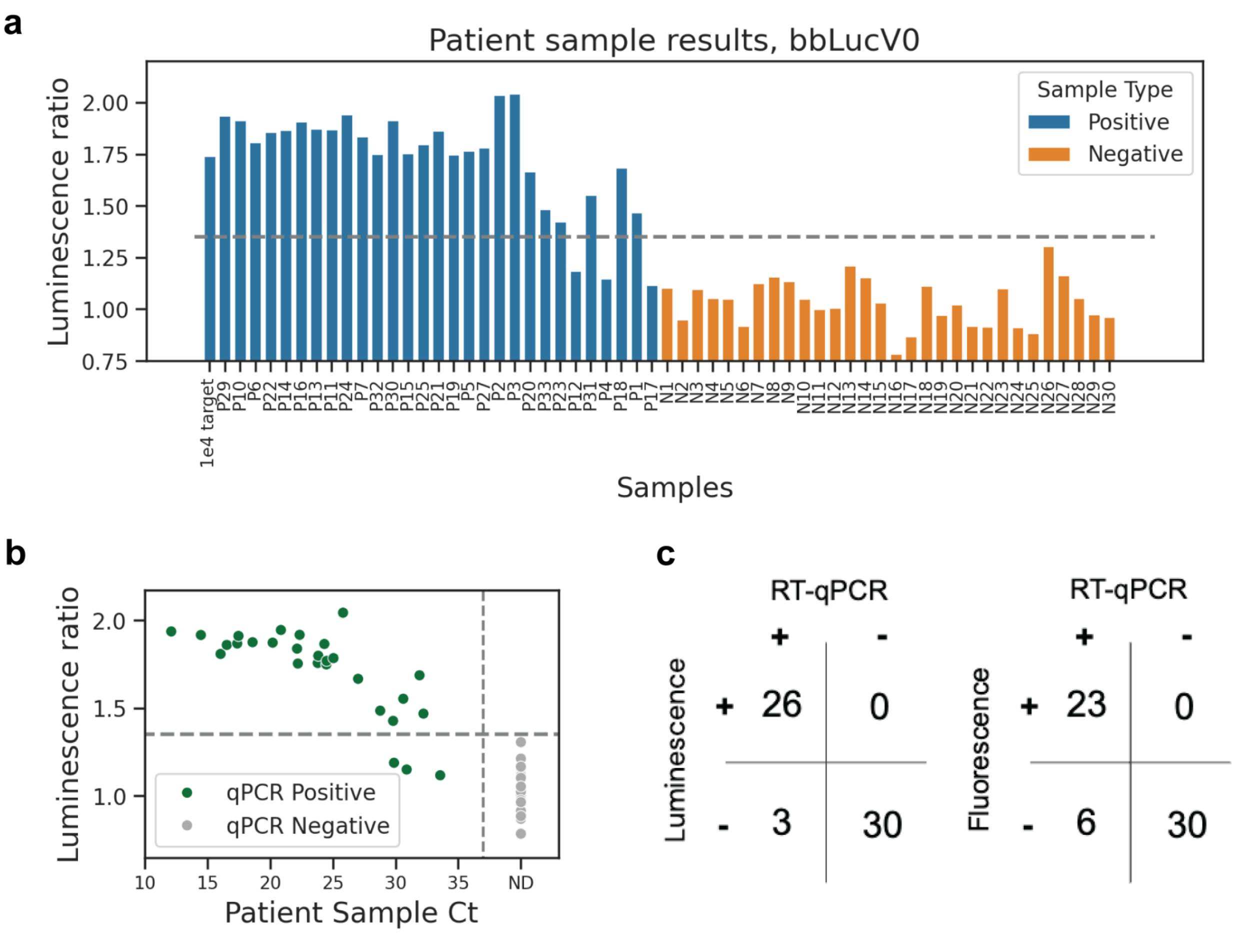

Supplementary Figure 13. We compared the performance of bbLucV0 SHINE and fluorescent SHINE to a gold-standard RT-qPCR on RNA extracted from 63 clinical swabs from suspected COVID-19 patients. Four out of 63 samples were negative for RNase P, a control gene that confirms adequate sample collection. Among the remaining 59 samples, 29 were RT-qPCR confirmed COVID-19 positive and 30 were confirmed COVID-19 negative. The remaining four were ruled inconclusive as they were negative for the Rnase P control test. bbLuc SHINE detected SARS-CoV-2 in 26 of the 29 positive samples (89.6% concurrence) compared to 23 of the 29 positive samples (79.3%) in fluorescent SHINE. Every positive sample detected by fluorescent SHINE was also detected as positive by our luminescent system, which additionally detected 3 high Ct (Ct > 28.5) samples that the fluorescent SHINE did not. Both fluorescent and luminescent SHINE correctly identified all 30 RT-qPCR negative samples as negative. (A) bbLucV0 SHINE scores for each patient sample (B) Comparison of bbLucV0 score and Ct value; (C) Confusion matrix comparing performance of bbLucV0 SHINE and fluorescence SHINE.

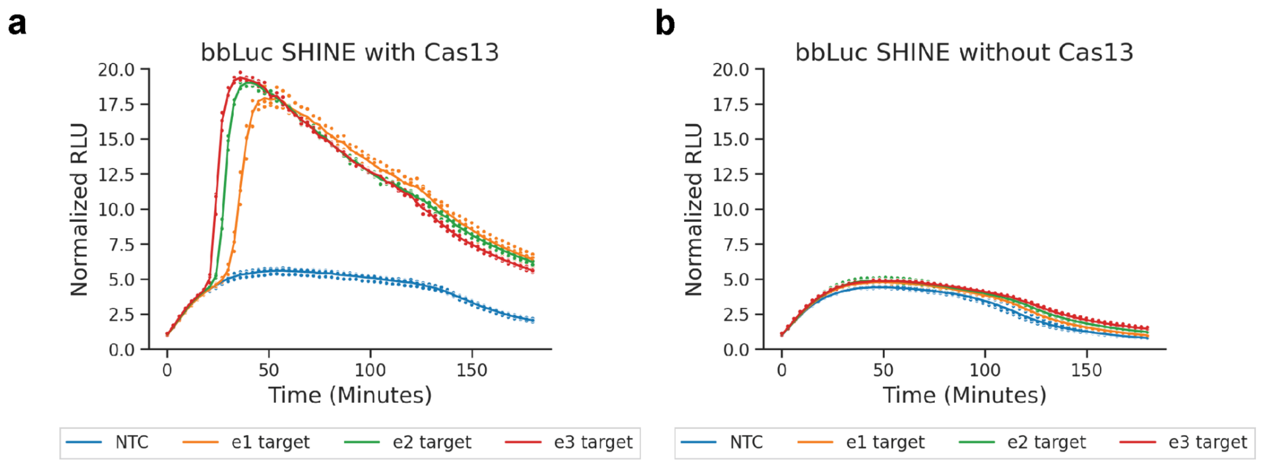

Supplementary Figure 14: Luminescent SHINE reactions (a) with and (b) without Cas13 were conducted to confirm that signal was due to Cas13-based cleavage of bbLuc reporter and not due to other effects such as contamination. Mastermixes were created in parallel with the same reagents and identical composition, except the replacement of Cas13 with excess water in (B).

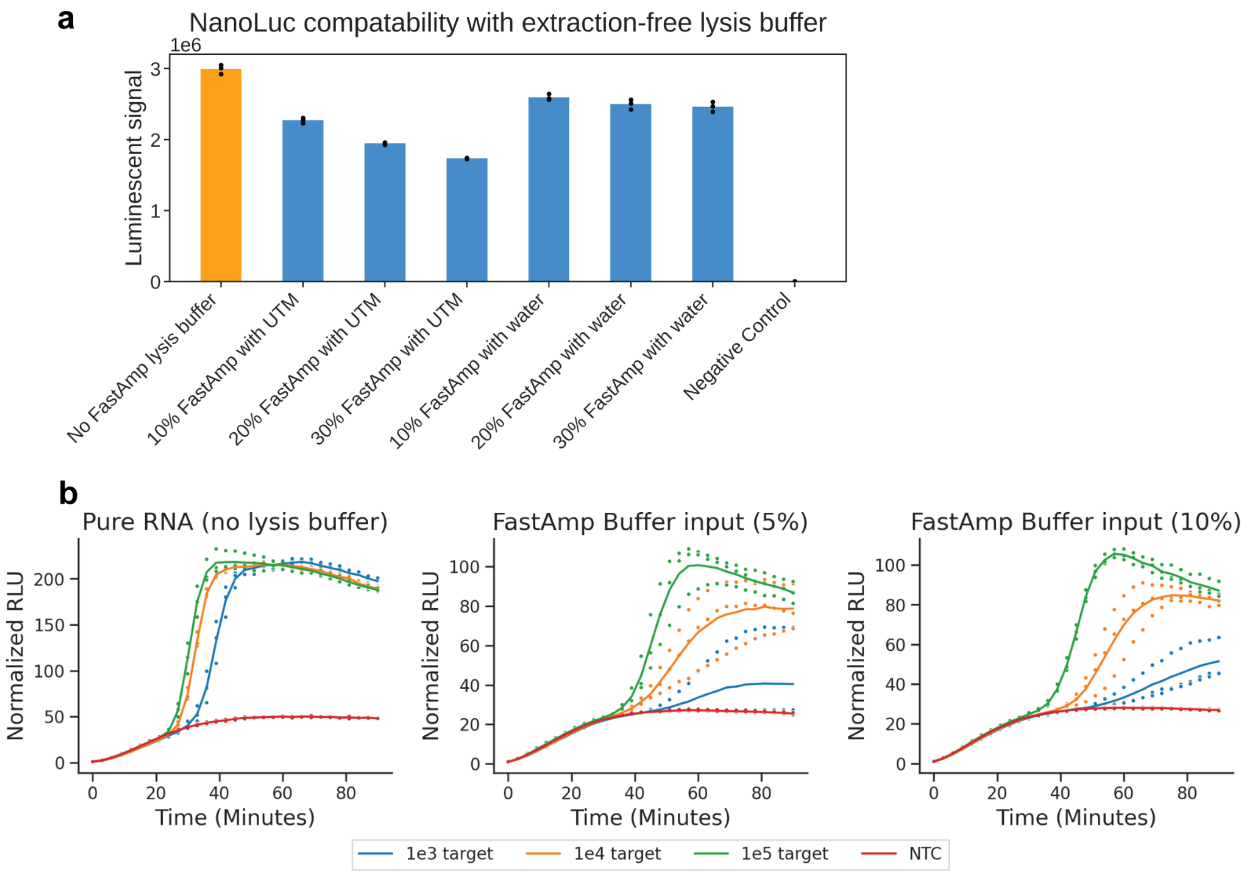

Supplementary Figure 15: (a) NanoLuc luminescent signal after 6-minute incubation with varying concentrations of FastAmp lysis buffer in UTM or water. Yellow bar shows control without lysis, N=3 replicates for all experiments. (b) Time-course detection of synthetic RNA targets (103-105 copies/µL) in mock samples containing UTM with FastAmp lysis buffer at 0%, 5%, or 10% of final reaction volume. Samples were prepared by combining RNA targets with UTM and FastAmp lysis buffer, incubating for 5 minutes at room temperature, then adding directly to bbLuc SHINE reaction. Detection performed under standard SHINE conditions with real-time luminescence monitoring over 90 minutes. NTC = no template control. Dotted lines represent individual replicates, solid lines represent mean values (N=3 technical replicates).

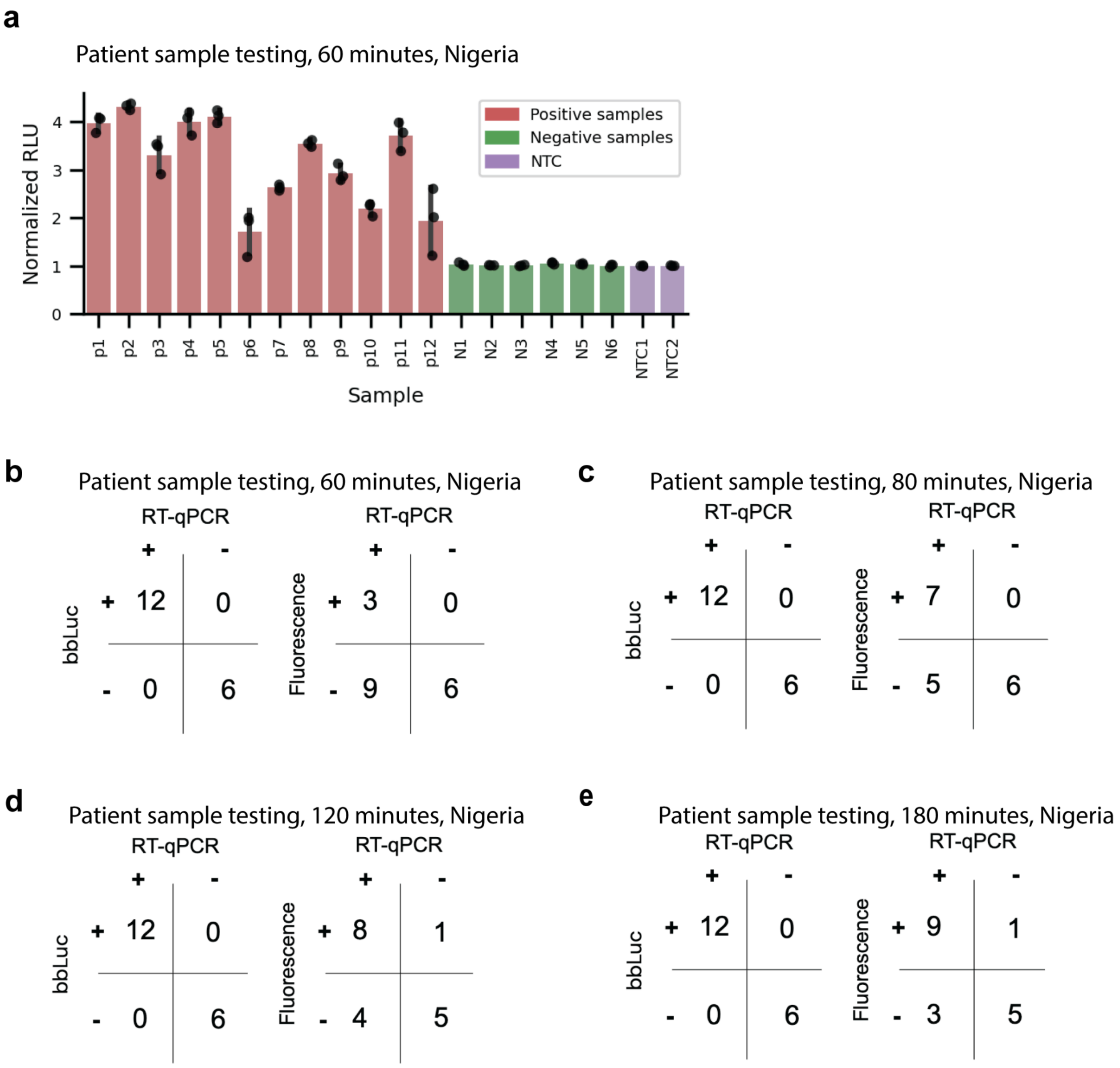

Supplementary Figure 16: We tested 18 patient samples (12 RT-qPCR positive and 6 RT-qPCR negative) using both the luminescent and fluorescent SHINE assays. (A) bbLuc readout after 60 minutes, showing 12 out of 12 RT-qPCR determined positive samples as positive and 6 out of 6 RT-qPCR samples as negative. (B-E) Confusion matrix comparisons of bbLuc and Fluorescent SHINE at 60, 80, 120, and 180 minutes. We note that because we used a portable luminometer for bbLuc testing in Nigeria, the bbLuc assay was moved from a 37° C incubator to the portable luminometer, and back, for each time point collected.

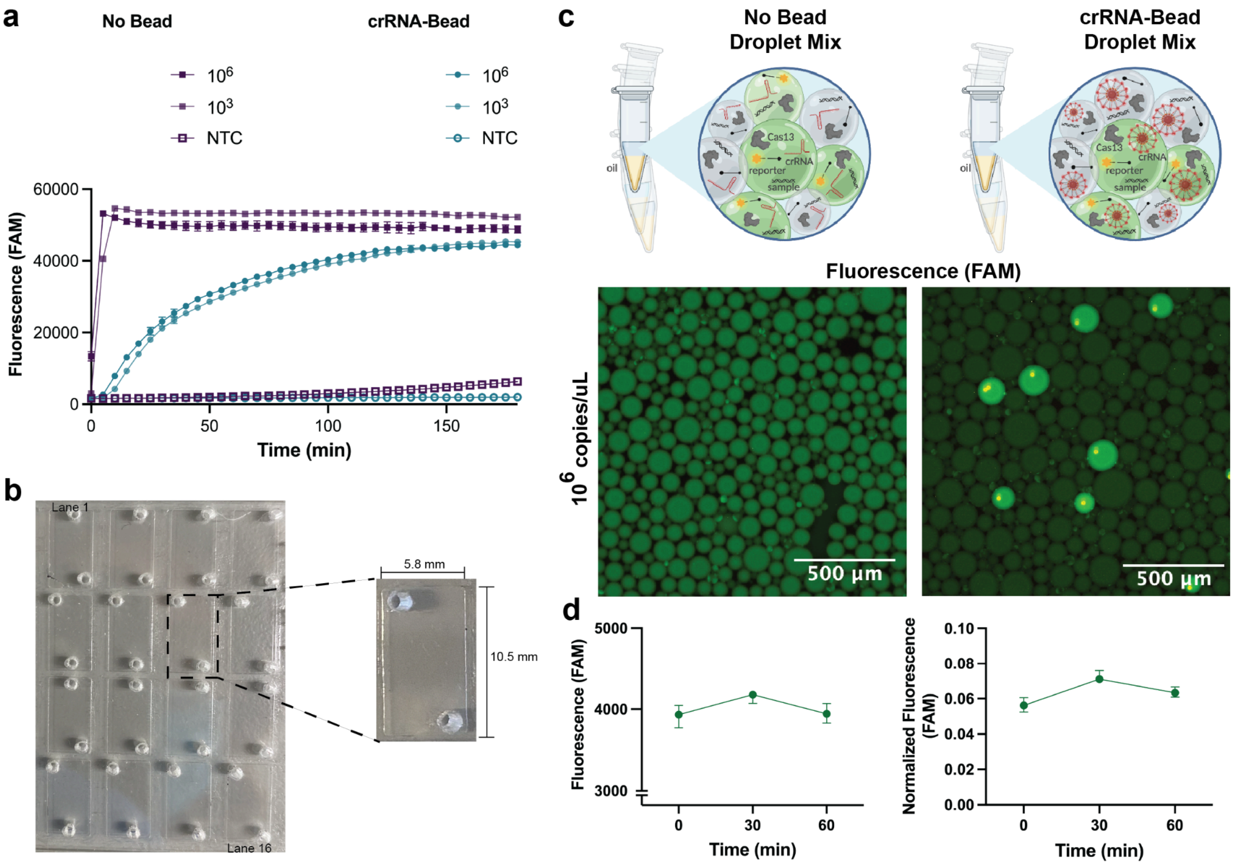

Supplementary Figure 17. Equipment-free droplet generation with color-coded crRNA beads for Cas13-based detection. a, Kinetics of SARS-CoV-2 at 10^6 and 10^3 copies/uL with SARS-CoV-2 crRNA either bound or not bound to a biotinylated bead. Fluorescence measured on the Cytation 5 plate reader. **b**, Image of flow cell with 5.8 x 10.5 mm lane dimensions that can be loaded using a multichannel pipette. Flow cells fabricated with 16 lanes, 25 x 75 mm, or 32 lanes, 50 x 75 mm. Shown as 16 lanes in **b**. **c**, Fluorescent images of droplets in the absence of beads (left) or presence of crRNA-beads (right) SARS-CoV-2 at 10^6 copies/uL. **d**, Fluorescence kinetics of SARS-CoV-2 crRNA in droplets with or without bead attachment and compared to signal from a no droplet control. Error bars denote 95% confidence intervals.

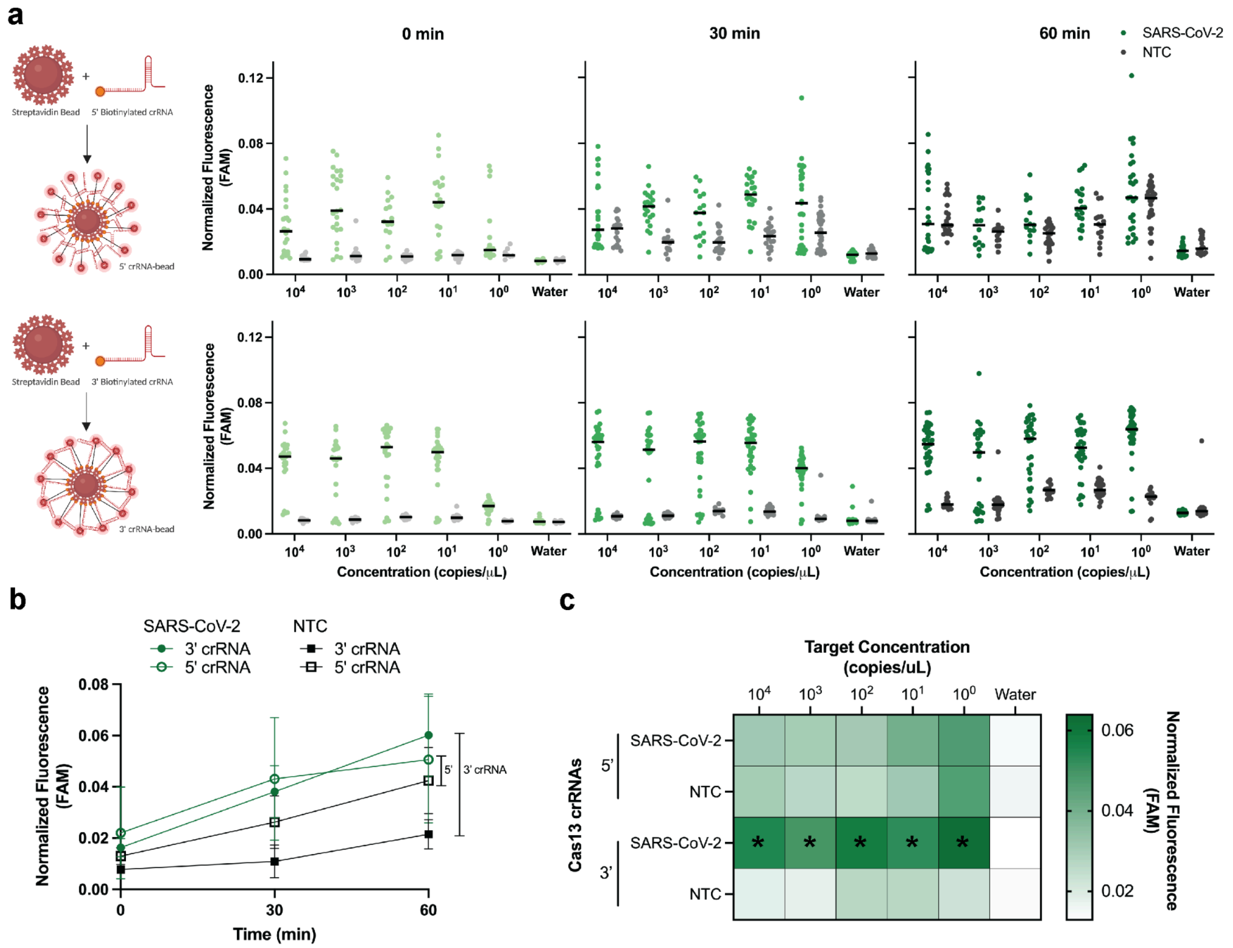

Supplementary Figure 18. Background signal reduction with 3' biotinylated crRNA-bead pools compared to 5' pools. a, Fluorescence across amplified SARS-CoV-2 dilution series from 10^4-10^0 copies/uL at 0, 30, and 60 min post-reaction initiation. Top: 5' biotinylated crRNA-bead pools; Bottom: 3' biotinylated crRNA-bead pools; Green: SARS-CoV-2; Gray: NTC. Bar at median fluorescence. **b**, Fluorescence kinetics of SARS-CoV-2 at 10^6 copies/uL from 3' and 5' biotinylated crRNA-bead pools. Green: SARS-CoV-2; Black: NTC; Closed points: 3' biotinylated crRNA; Open points: 5' biotinylated crRNA. **c**, Heatmap of median SARS-CoV-2 and NTC fluorescence at 60 min post-reaction initiation from **a**. Asterisk (*) represents positive signal detected above threshold. Error bars denote 95% confidence intervals.

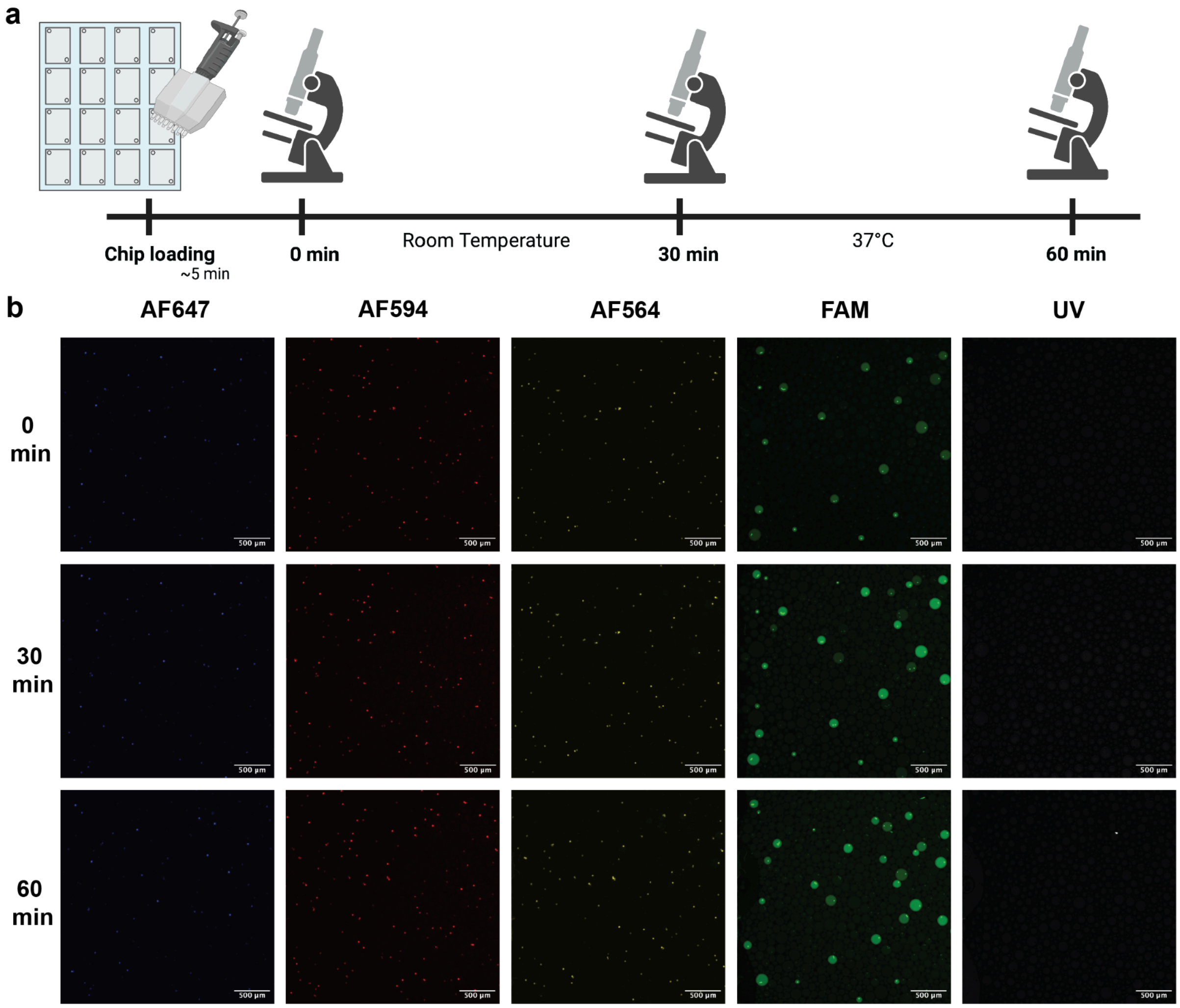

Supplementary Figure 19. Dual SARS-CoV-2 and RNase P fluorescence imaging over time. a, Schematic of bbCARMEN flow cell imaging up to 60 min post-reaction initiation. **b**, Fluorescent images at 0, 30, and 60 min post-reaction in 4 different fluorescent channels. Synthetic SARS-CoV-2 RNA at 10^6 copies/uL was spiked into RNase P. Blue: AF647; Red: AF594; Yellow: AF564; Green: FAM. AF647: Semrock LF635-B; AF594: Semrock 3FF03-575/25-25 and FF01-615/24-25; AF546: Semrock SpGold-B; FAM: Semrock GFP-1828A. Scale bar: 500 µm.

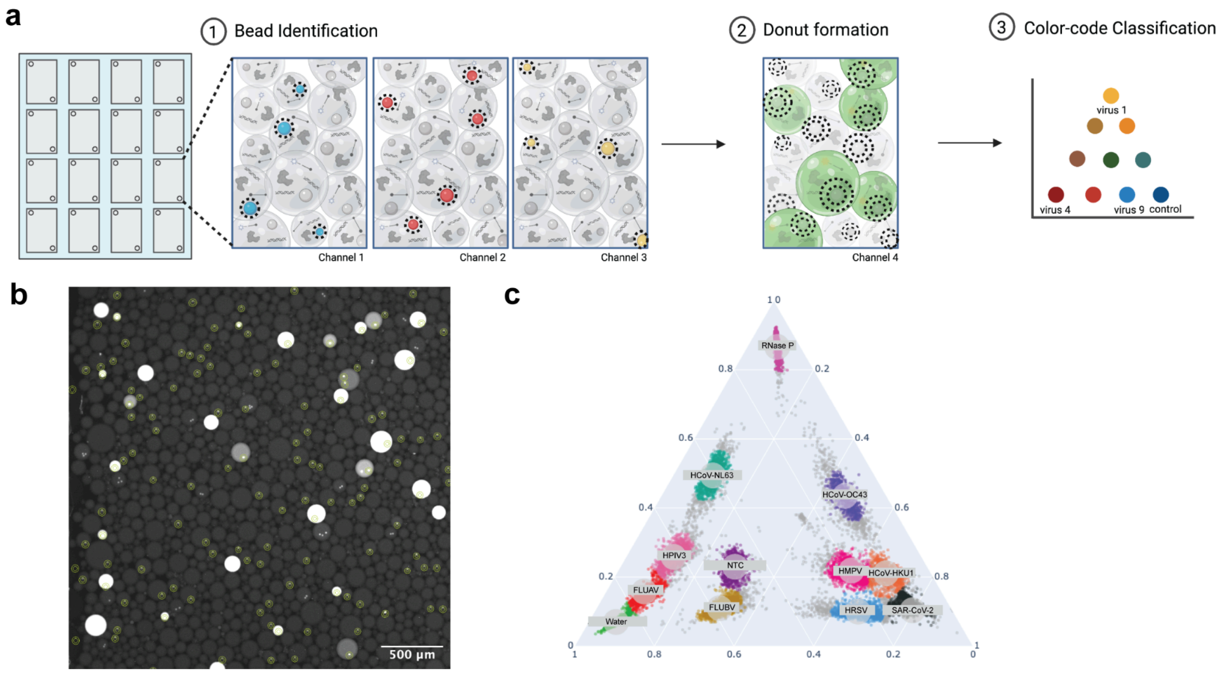

Supplementary Figure 20. Dual SARS-CoV-2 and RNase P fluorescence imaging over time. a, Schematic of bbCARMEN flow cell imaging up to 60 min post-reaction initiation. b, Fluorescent images at 0, 30, and 60 min post-reaction in 4 different fluorescent channels. Synthetic SARS-CoV-2 RNA at 10^6 copies/uL was spiked into RNase P. Blue: AF647; Red: AF594; Yellow: AF564; Green: FAM. AF647: Semrock LF635-B; AF594: Semrock 3FF03-575/25-25 and FF01-615/24-25; AF546: Semrock SpGold-B; FAM: Semrock GFP-1828A. Scale bar: 500 µm.

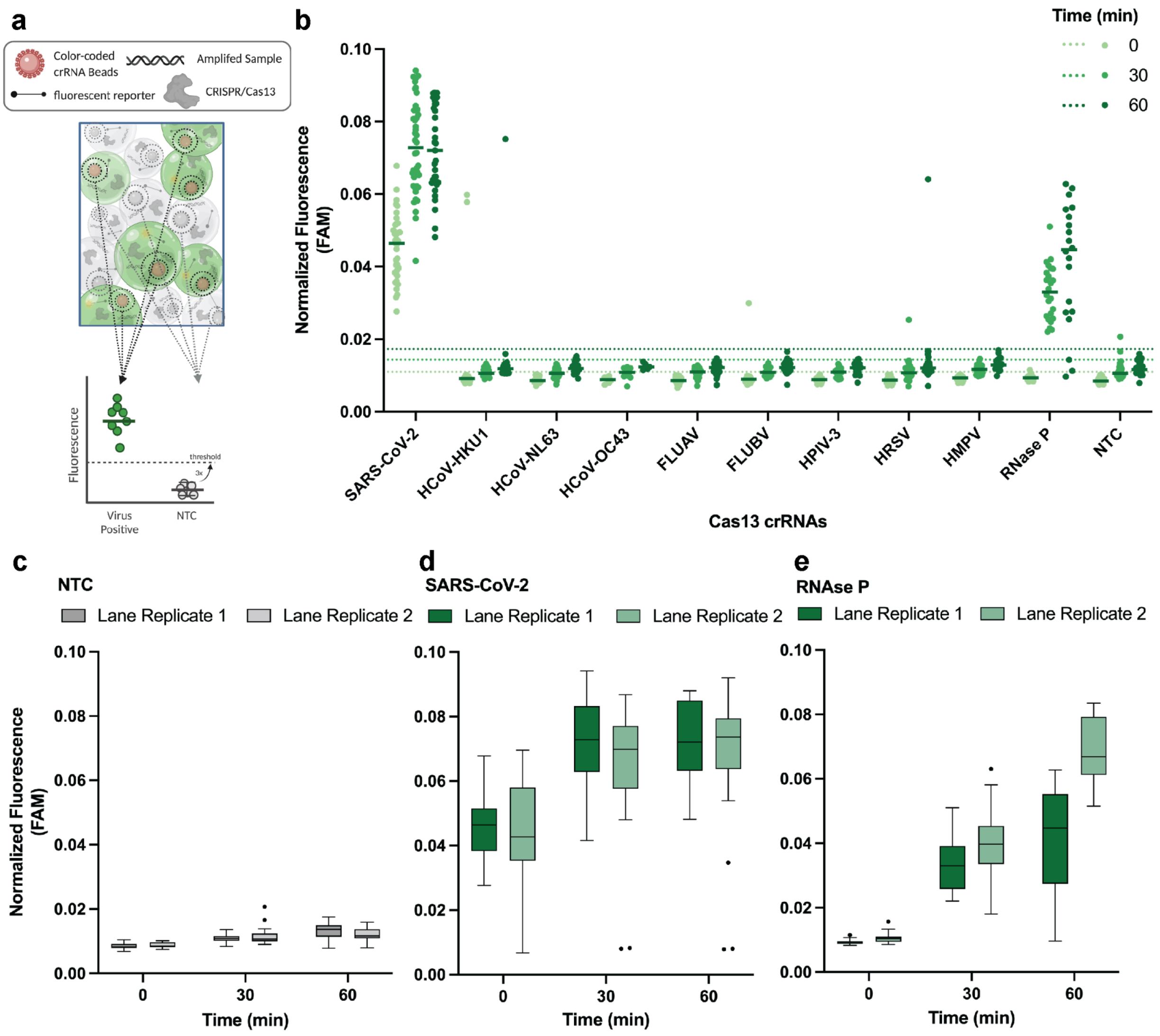

Supplementary Figure 21. Characterization of SARS-CoV-2 FAM fluorescence signal across all bead and lane replicates. a, Schematic of fluorescence values derived from signals within the donuts formed around a bead within a droplet. **b**, FAM fluorescence within donuts of each color-coded crRNA bead population that make up RVP at 0, 30, and 60 min post-reaction initiation. Bar at median fluorescence. Thresholds shown as dashed lines at each time point calculated as 3x the standard deviation of the NTC. **c-e**, Tukey box and whiskers plot of fluorescence values across lane replicates at 0, 30, and 60 min post-reaction initiation. Outliers represented as single points. **c**, NTC fluorescence. **d**, SARS-CoV-2 fluorescence. **e**, RNase P fluorescence.

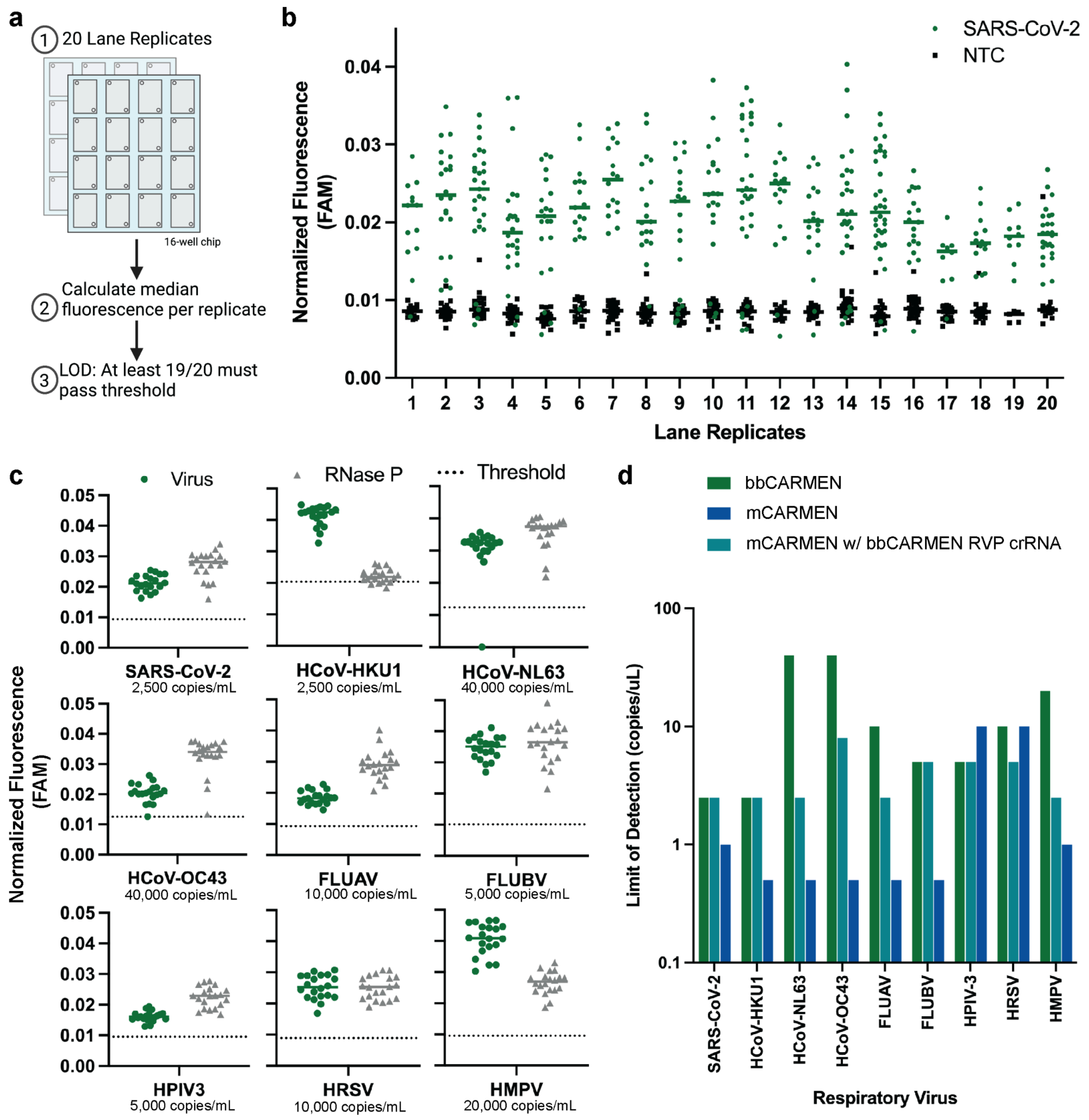

Supplementary Figure 22. RVP limit of detection evaluation on bbCARMEN. a, Schematic of LOD testing with bbCARMEN. **b**, Fluorescence of SARS-CoV-2, 2.5 copies/uL, and NTC for each of the 20 technical replicates. Individual points represent signal from a single droplet with a bar at median fluorescence. Green: SARS-CoV-2; Black: NTC. **c**, Median fluorescence (n=20) at the LOD for each of the 9 viruses on RVP as established by spiking synthetic RNA into negative control RNA. Green: virus; Gray: RNase P; Dashed line: Threshold derived from NTC. **d**, Comparison of RVP LODs across the CARMEN technologies. Green: CARMEN v2 RVP assay; Teal: CARMEN v2 RVP assay detectable by mCARMEN; Blue: mCARMEN RVP assay

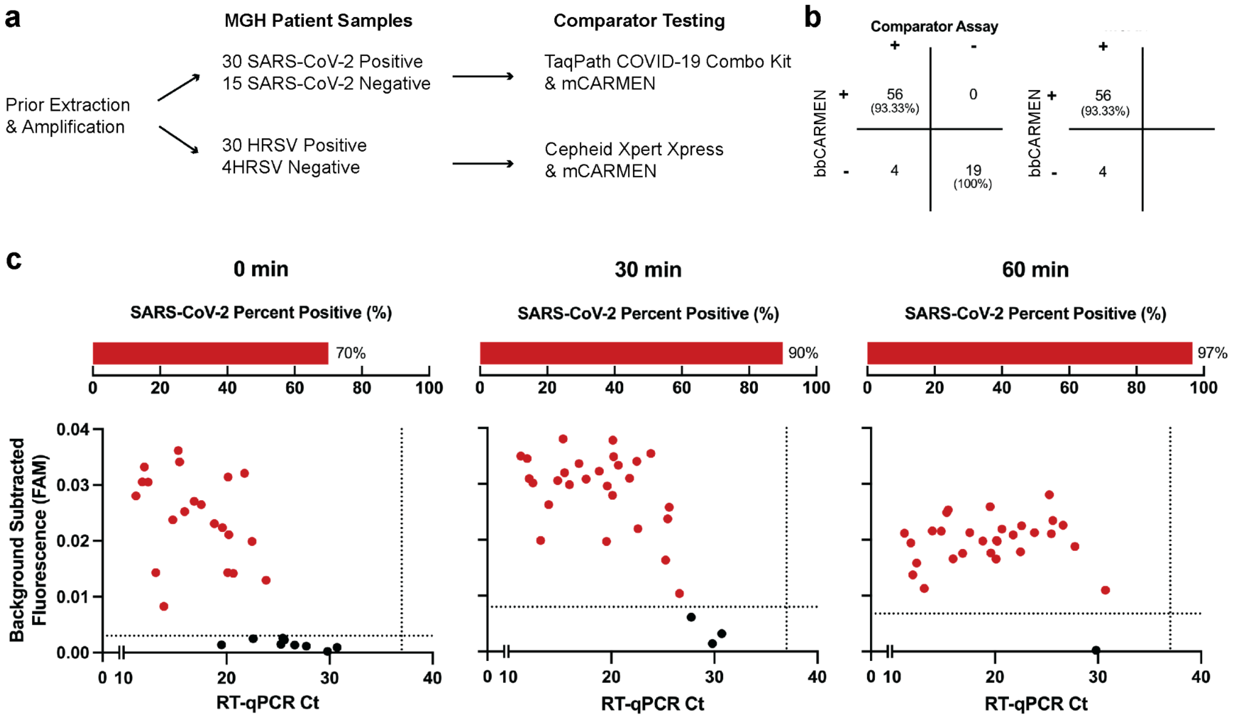

Supplementary Figure 23. Comparison of bbCARMEN detection results to results collected from comparator assays. a, Schematic of patient sample testing, with 79 patient samples tested, including 45 SARS-CoV-2 samples (30 positive, 15 negative) and 34 HRSV samples (30 positive, 4 negative). **b**, Concordance of bbCARMEN and bbCARMEN for 79 patient samples. **c**, Scatter plot of scaled normalized fluorescent values compared to viral Ct values detected by RT-qPCR at 0 min, 30 min, and 60 min timepoints for the positive SARS-CoV-2 samples.

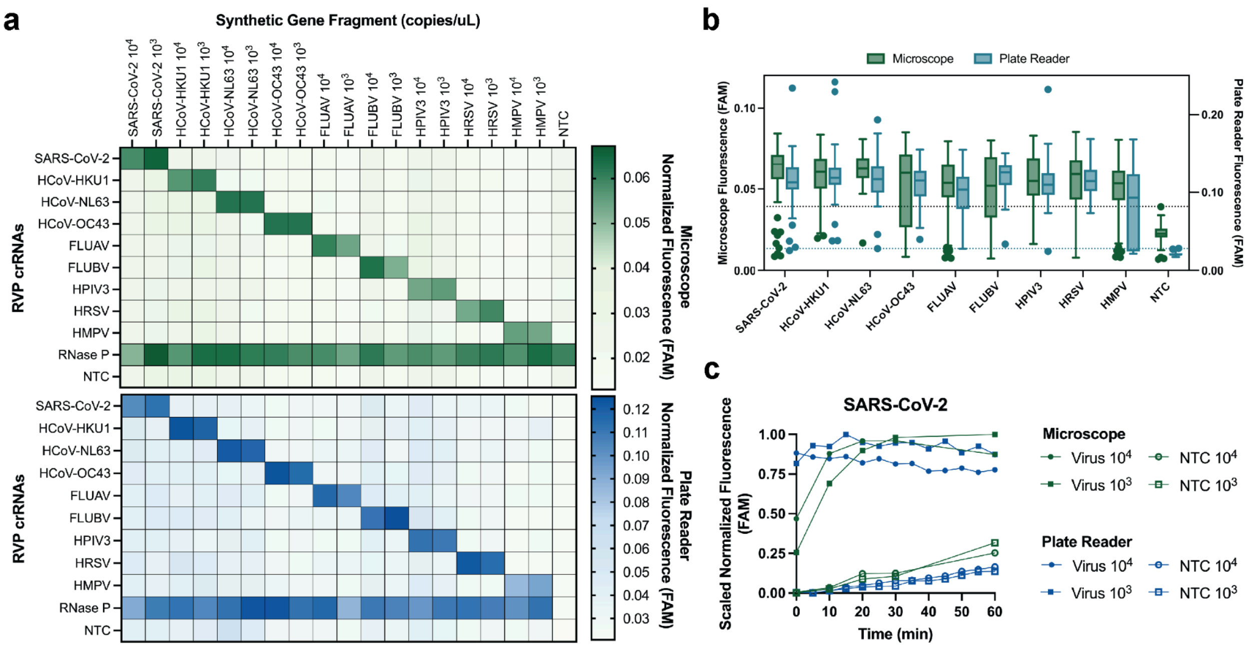

Supplementary Figure 24: Comparison of bbCARMEN fluorescent results from two readout platforms. **a**, Heatmaps at 60 min post-reaction initiation. **b**, Tukey box and whisker plots at 10^3 copies/uL 60 min post-reaction initiation; dashed line: 3 std dev above the median NTC. **c**, Kinetic curves of runs with 10^4 and 10^3 copies of SARS-CoV-2 on the microscope and plate reader-based platforms, including scrambled crRNA NTC values for each input sample.

Supplementary Tables

Table 1 – oligos for bbLuc

Oligo name	Sequence
HaloLigand-based linker, 7U	/5Biosg/rUrUrU rUrUrU rU/HaloLigand/
HaloLigand-based linker, 14U	/5Biosg/rUrUrU rUrUrU rUrUrU rUrUrU rUrU/HaloLigand/
HaloLigand-based linker, 21U	/5Biosg/rUrUrU rUrUrU rUrUrU rUrUrU rUrUrU rUrUrU rUrUrU/HaloLigand/
DNA-RNA linker (internal name: SPAAC linker, 21U, 33 dNTPs)	/5Biosg/rUrUrU rUrUrU rUrUrU rUrUrU rUrUrU rUrUrU rUrUrU TTA TTA TTA TTA TTA TTA GGA GGA GCA CGA GGA/3DBCO/
21 Uracil linker	/5Biosg/rUrUrU rUrUrU rUrUrU rUrUrU rUrUrU rUrUrU rUrUrU/3DBCO/
9 Uracil linker	/5Biosg/rUrUrU rUrUrU rUrUrU /3DBCO/
Maleimide-thiol 21U Thiol	/5Biosg/rUrUrU rUrUrU rUrUrU rUrUrU rUrUrU rUrUrU rUrUrU/3ThioMC3-D/
Thiol 21U 7-hexapeg	/5Biosg/rUrU rUrUrU rUrUrU rUrUrU rUrUrU rUrUrU rUrUrU rU/iSp18//iSp18/ /iSp18//iSp18//iSp18/ /iSp18//iSp18//3ThioMC3-D/
Thiol 2-hexapeg 14 U	/5Biosg//iSp18//iSp18//rUrUrU rUrUrU rUrUrU rUrUrU rUrU/3ThioMC3-D/
Thiol 3-hexapeg 10 U	/5Biosg//iSp18//iSp18//iSp18//rUrUrU rUrUrU rUrUrU rU/3ThioMC3-D/
Thiol 4-hexapeg 6U	/5Biosg//iSp18//iSp18//iSp18//iSp18//rUrUrU rUrUrU/3ThioMC3-D/
SPAAC 2-hexapeg 14 U	/5Biosg//iSp18//iSp18/rUrUrU rUrUrU rUrUrU rUrUrU rUrU/3DBCON/
SPAAC 3-hexapeg 10 U	/5Biosg//iSp18//iSp18//iSp18/rUrUrU rUrUrU rUrUrU rU/3DBCON/
SPAAC 4-hexapeg 6U	/5Biosg//iSp18//iSp18//iSp18//iSp18/rUrUrU rUrUrU/3DBCON/
SPAAC 4-hexapeg 14 U	/5Biosg//iSp18//iSp18//iSp18//iSp18/rUrUrU rUrUrU rUrUrU rUrUrU rUrU/3DBCON/
Thiol 2-hexapeg 21 U	/5Biosg//iSp18//iSp18//rUrUrU rUrUrU rUrUrU rUrUrU rUrUrU rUrUrU rUrUrU/3ThioMC3-D/
Thiol 4-hexapeg 21 U	/5Biosg//iSp18//iSp18//iSp18//iSp18//rUrUrU rUrUrU rUrUrU rUrUrU rUrUrU rUrUrU rUrUrU/3ThioMC3-D/

Spike 69/70 gBlock	gaaatTAATACGACTCACTATAggTGACAAAGTTTTCAGATCCTCAGTTTTACATTCAACTCAGGACTTGTTCTTACCTTTCTTTTCCAATGTTACTTGGTTCCATGCTATACATGTCTCTGGGACCAATGGTACTAAGAGGTTTGATAACCCTGTCCTACCATTTAATGATGGTGTTTATTTTGCTTCCACTGAGAAGTCTAACATAATAAGAGGCTGGATTTTTGGTACTACTTTAGATTCGAAGACCCAGTCCCTACTTATTGTTAATAACGCTACTAATGTTGTTATTAAAGTCTGTGAATTTC
Spike RPA primer forward	gaaatTAATACGACTCACTATAgggCAACTCAGGACTTGTTCTTACCTTTCTTTTCC
Spike RPA primer reverse	AAGCAAAATAAACACCATCATTAAAT
Spike crRNA 69/70	rGrArU rUrUrA rGrArC rUrArC rCrCrC rArArA rArArC rGrArA rGrGrG rGrArC rUrArA rArArC rArCrA rGrGrG rUrUrA rUrCrA rArArC rCrUrC rUrUrA rGrUrA rCrCrA rU
FAM_7U_Reprter – excitation @ 495nM, emission @ 520nM	/56-FAM/rUrUrUrUrUrUrU/3IABkFQ/

Table 2 – oligos for bbCARMEN

FAM_7U_reporter (reporter oligo)	/56-FAM/rUrUrUrUrUrUrU/3IABkFQ/
Dye001_5'AF546_5C_3'Bio (bead dye fluorophore) – excitation @ 561nM, emission @ 572nM	/5Alex546N/CC CCC /3Bio/
Dye002_5'AF594_5C_3'Bio (bead dye fluorophore) – excitation @ 590nM, emission @ 618nM	/5Alex594N/CC CCC /3Bio/
Dye003_5'AF647_5C_3'Bio (bead dye fluorophore) – excitation @ 650nM, emission @ 671nM	/5Alex647N/CC CCC /3Bio/
SARS-CoV-2 crRNA (biotinylated)	rGrArUrUrUrArGrArCrUrArCrCrCrCrArArArArArCrGrArArGrGrGrGrArCrUrArArArArCrCrUrArArArArCrUrArUrUrCrArCrUrUrCrArArUrArGrUrCrUrGrArA/3Bio/
HCoV-HKU1 crRNA (biotinylated)	rGrArUrUrUrArGrArCrUrArCrCrCrCrArArArArArCrGrArArGrGrGrGrArCrUrArArArArCrArArUrArUrGrArUrUrArCrCrArUrUrArCrCrArCrArArArArArUrUrA/3Bio/
HCoV-NL63 crRNA (biotinylated)	rGrArUrUrUrArGrArCrUrArCrCrCrCrArArArArArCrGrArArGrGrGrGrArCrUrArArArArCrUrUrArArUrArGrUrUrUrCrArGrCrCrGrCrArArArGrArGrUrCrUrArA/3Bio/
HCoV-OC43 (BetaCoV) crRNA (biotinylated)	rGrArUrUrUrArGrArCrUrArCrCrCrCrArArArArArCrGrArArGrGrGrGrArCrUrArArArArCrUrGrUrUrGrUrArArCrGrCrCrCrUrUrArUrArArUrArGrArCrCrUrUrA/3Bio/

HPIV3 crRNA (biotinylated)	rGrArUrUrUrArGrArCrUrArCrCrCrCrArArArArArCrGrArArGrGrGrGrArCrUrArArArArCrGrUrCrGrCrArUrUrUrCrCrCrCrUrCrArArUrArGrArGrUrCrCrUrU/3Bio/
FluA crRNA (biotinylated)	rGrArUrUrUrArGrArCrUrArCrCrCrCrArArArArArCrGrArArGrGrGrGrArCrUrArArArArCrArArArArArGrCrUrUrGrUrGrArArUrUrCrArArArUrGrUrCrCrCrUrG/3Bio/
FluB crRNA (biotinylated)	rGrArUrUrUrArGrArCrUrArCrCrCrCrArArArArArCrGrArArGrGrGrGrArCrUrArArArArCrArCrUrArArArCrArGrArUrCrArGrGrArCrArArGrGrUrArUrUrUrGrG/3Bio/
HMPV crRNA (biotinylated)	rGrArUrUrUrArGrArCrUrArCrCrCrCrArArArArArCrGrArArGrGrGrGrArCrUrArArArArCrGrUrCrGrCrArArArArGrArCrArUrGrGrUrCrUrCrCrUrCrUrUrGrUrU/3Bio/
HRSV crRNA (biotinylated)	rGrArUrUrUrArGrArCrUrArCrCrCrCrArArArArArCrGrArArGrGrGrGrArCrUrArArArArCrGrUrCrUrUrUrUrCrUrArGrGrArCrArUrUrGrUrArUrUrGrArArCrA/3Bio/
RNaseP crRNA (biotinylated)	rGrArUrUrUrArGrArCrUrArCrCrCrCrArArArArArCrGrArArGrGrGrGrArCrUrArArArArCrUrCrCrGrArGrUrCrArGrUrGrGrCrUrCrCrCrGrUrGrUrGrUrCrGrGrU/3Bio/
Scrambled 1 crRNA (biotinylated)	rGrArUrUrUrArGrArCrUrArCrCrCrCrArArArArArCrGrArArGrGrGrGrArCrUrArArArArCrArCrGrUrCrUrArArUrArCrGrArUrArCrArUrCrArUrUrArCrArUrArU/3Bio/
Scrambled 2 crRNA (biotinylated)	rGrArUrUrUrArGrArCrUrArCrCrCrCrArArArArArCrGrArArGrGrGrGrArCrUrArArArArCrGrUrGrCrGrCrCrGrUrUrGrGrCrUrCrGrUrGrUrArGrCrArGrUrUrCrC/3Bio/
SARS-CoV-2 forward primer	gaaatTAATACGACTCACTATAgggCAATTAGAGATGGAACTTACACC
HCoV-HKU1 forward primer	gaaatTAATACGACTCACTATAgggGTGTGTTAAAAGTCAATCTCCTCG
HCoV-NL63 forward primer	gaaatTAATACGACTCACTATAgggACTTGCTAATGATGTTAAAGATACAC
HCoV-OC43 (BetaCoV) forward primer	gaaatTAATACGACTCACTATAgggGCTAAGAATGAGAGTAGTTCATTG
HPIV3 forward primer	gaaatTAATACGACTCACTATAgggTGATCTCAATGAAATTAGAAAGATGG
FluA forward primer	gaaatTAATACGACTCACTATAgggGAGCAAAAAGAAGTCCTATATAAATAAG
FluB forward primer	gaaatTAATACGACTCACTATAgggCAAGCAAAACAAAAAGACTAAAGGC
HMPV forward primer	gaaatTAATACGACTCACTATAgggACCCAAATGAGAAAGACTGTG
HRSV forward primer	gaaatTAATACGACTCACTATAgggCTTCACGAAGGCTCCA

	CATA
RNaseP forward primer	gaaatTAATACGACTCACTATAgggTTGATGAGCTGGAGCCA
SARS-CoV-2 reverse primer	CTTTTTAGCTTCTTCCACAATGTC
HCoV-HKU1 reverse primer	AACCATAAGGAGCATTTTGAAC
HCoV-NL63 reverse primer	GACTTAACACTCTCTTCTTTAGCT
HCoV-OC43 (BetaCoV) reverse primer	ATTTACAGCACTAGAACTTTCATG
HPIV3 reverse primer	CTGATATCTCGCTTGGAACATCTGCAG
FluA reverse primer	AATTAGCCACAAATCCATAGCG
FluB reverse primer	TGTTTCTTCATTATATCTTTCTAATGGTAT
HMPV reverse primer	GCAACATTAATTCCTGCTGCT
HRSV reverse primer	CCCATATTGTTAGTGATGCAGG
RNaseP reverse primer	ATGTGGATGGCTGAGTTGTT
SARS-CoV-2 gBlock	GCACCCATATTGTTAGTG
HCoV-HKU1 gBlock	gaaatTAATACGACTCACTATAgggATGCTCTTCTTTCTATTCAGAATGGTTTTAGTGCTACCAACTCTGCACTTGCTAAAATACAAAGTGTTGTTAATTCTAATGCTCAAGCACTTAATAGTTTGTTACAGCAATTATTTAATAAATTTGGTGCAATTAGTTCTTCTTTACAAGAAATTTTATCTCGTCTCGATGCTTTAGAGGCTCAGGTTCAGATTGATAGGCTTATTAATGGTCGTTTAACTGCTTTAAATGCTTATGTCTCTCAACAGCTTAGTGATATTTCTCTTGTAAAATTTGGTGCTGCTTTAGCTATGGAGAAGGTTAATGAGTGTGTTAAAAGTCAATCTCCTCGTATTAATTTTTGTGGTAATGGTAATCATATTTTGTCATTAGTTCAAAATGCTCCTTATGGTTTGTTGTTTATGCATTTTAGTTATAAACCTATTTCTTTTAAAACTGTTTTAGTAAGTCCTGGTTTGTGTATATCAGGTGATGTAGGTATTGCACCTAAACAAGGGTAT
HCoV-NL63 gBlock	gaaatTAATACGACTCACTATAgggACGTTATGTGTCTTTAGCTATTGATGCATACCCTCTTTCAAAACACCCTAATTCTGAATATCGTAAGGTTTTTTACGTATTACTTGATTGGGTTAAGCATCTTAACAAAAATTTGAATGAGGGTGTTCTTGAATCTTTTTCTGTTACACTTCTTGATAATCAAGAAGATAAGTTTTGGTGTGAAGATTTTTATGCTAGTATGTATGAAAATTCTACAATATTGCAAGCTGCTGGTTTATGTGTTGTTTGTGGTTCACAAACTGTACTTCGTTGTGGTGATTGTCTGCGTAAGCCTATGTTGTGCACTAAATGCGCATATGATCATGTATTTGGTACCGACCACAAGTTTATTTTGGCTATAACACCGTATGTATGTAATGCATCAGGTTGTGGTGTTAGTGATGTCAAAAAATTGTATCTTGGTGGTTTGAATTACTATTGTACAAATCATAAACCACAGTTGTCTTTTCCATTATGTTCAGCTGGTAATATATTTGGTTTATATAAAAATTCAGCAACTGGTTCCTTAGATGT

	TGAAGTTTTTAATAGGCTTGCAACGTCTGATTGGACTGA TGTTAGGGACTATAAACTTGCTAATGATGTTAAAGATACA CTTAGACTCTTTGCGGCTGAAACTATTAAAGCTAAAGAA GAGAGTGTTAAGTCTTCTTATGCTTTTG
HCoV-OC43 (BetaCoV) gBlock	gaaatTAATACGACTCACTATAgggGTTGTAGATGAAGTTAG CATGCTTACCAATTATGAGCTTTCTGTTATTAATGCTCGT ATTCGTGCTAAGCATTATGTTTATATTGGTGATCCTGCTC AATTGCCAGCACCACGTGTGTTATTGAGCAAGGGTACA CTTGAACCTAAATATTTTAACACTGTTACTAAGCTCATGT GTTGCTTAGGGCCAGACATTTTTCTTGGTACATGTTATAG ATGTCCTAAGGAAATTGTTGATACAGTGTCCGCCTTGGT TTATGAAAATAAGCTTAAGGCTAAGAATGAGAGTAGTTCA TTGTGTTTTAAGGTCTATTATAAGGGCGTTACAACACATG AAAGTTCTAGTGCTGTAAATATGCAGCAGATTTATTTGAT TAATAAGTTTTTGAAGGCTAACCCTTTGTGGCATAAAGCT GTTTTTATTAGCCCATATAATAGTCAGAACTTTGCAGCTA AGCGTGTTTTGGGTTTACAAACCCAAACCGTGGATTCT GCTCAAGG
HPIV3 gBlock	gaaatTAATACGACTCACTATAgggACCATCTGTCAACCAG AAATCAAACCAACAGAAACAAGTGAAAAAGATAGTGGAT CAACTGACAAAAATAGACAGTCTGGGTCATCACACGAAT GTACAACAGAAGCAAAAGATAGAAATATTGATCAGGAAA CTGTACAGAGAGGACCTGGGAGAAGAGGCAGCTCAGA TAGTAGAGCTGAGACTGTGGTCTCTGGAGGAATCTCCA GAAGCATCACAGATTCTAAAAATGGAACCCAAAACACGG AGAATATTGATCTCAATGAAATTAGAAAGATGGATAAGGA CTCTATTGAGGGGAAAATGCGACAATCTGCAGATGTTCC AAGCGAGATATCAGGAAGTGATGGCATATTTACAACAGA ACAAAGTAGAAACAGTGATCATGGAAGAAGCTTGGAATC TATCGGTACACCTGATACAAGATCAATAAGTGTTGTTACT GCTGCAACACCAGATGATGAAGAAGAAATACTAATGAGA AATAGTAGGATGAAGAA
FluA gBlock	gaaatTAATACGACTCACTATAgggTGAATCAACAAGGAAG AAAATTGAGAAGATAAGGCCTCTTTTAATGGATGGCACA GCATCACTGAGTCCTGGGATGATGATGGGCATGTTCAA CATGCTAAGTACAGTCTTGGGAGTCTCGATACTGAATCT TGGACAAAAGAAATACACCAAGACAACATACTGGTGGG ATGGGCTCCAATCATCCGACGATTTTGCTCTCATAGTGA ATGCACCAAACCATGAAGGAATACAAGCAGGAGTGGAC AGATTCTACAGGACCTGCAAATTAGTGGGAATCAACATG AGCAAAAAGAAGTCCTATATAAATAAGACAGGGACATTT GAATTCACAAGCTTTTTTTATCGCTATGGATTTGTGGCTA ATTTTAGCATGGAGCTACCCAGCTTTGGAGTGTCTGGA GTAAATGAATCAGCTGACATGAGTATTGGAGTAACAGTG ATAAAGAACAACATGATAAACAATGACCTTGGACCTGCA ACGGCTCAGATGGCTCTTC

FluB gBlock	gaaatTAATACGACTCACTATAgggCAAGCAAAACAAAAAG ACTAAAGGCCCAAATACCTTGTCCTGATCTGTTTAGTATA CCATTAGAAAGATATAATGAAGAAACAAGGGCAAAATTG AAGAAGCTAAAACCATTCTTCAATGAAGAAGGAACTGCA TCTTTGTCACCTGGGATGATGATGGGAATGTTTAATATG CTATCTACCGTGTTGGGAGTAGCTGCACTAGGTATCAAG AACATTGGAAACAAAGAATACCTATGGGATGGACTGCAA TCTTCTGATGATTTTGCTCTATTTGTTAATGCAAAGGATG AAGAAACATGTATGGAAGGAATAAACGACTTTTACCGAA CATGTAAATTATTGGGAATAAACATGAGCAAAAAGAAAAG TTACTGTAATGAGACTGGAATGTTTGAATTTACAAGCATG TTCTACAGAGATGGATTTGTATCTAATTTTGCAATGGAAC TCCCTTCGTTTGGGGTTGCTGGAGTAAATGAATCAGCA GATATGGCAATA
HMPV gBlock	gaaatTAATACGACTCACTATAgggGAGAAGACCAAGGGTG GTATTGTCAGAATGCAGGGTCAACTGTTTACTACCCAAA TGAGAAAGACTGTGAAACAAGAGGAGACCATGTCTTTT GCGACACAGCAGCAGGAATTAATGTTGCTGAGCAATCA AAGGAGTGCAACATCAACATATCCACTACAAATTACCCAT GCAAAGTCAGCACAGGAAGACATCCTATCA
HRSV gBlock	gaaatTAATACGACTCACTATAgggGGGGCAAATATGGAAA CATACGTGAACAAACTTCACGAAGGCTCCACATACACAG CTGCTGTTCAATACAATGTCCTAGAAAAAGACGATGATC CTGCATCACTTACAATATGGGTGCCCATGTTCCAATCAT CCATGCCAGCAGATTTACTTATAAAAGAACTAGCTAATGT CAACATACTAGTGAAACAAATATCCACA
RNaseP gBlock	gaaatTAATACGACTCACTATAgggTCCTTGCAGGTGGCTG CCAATACCTCCACCGTGGAGCTTGTTGATGAGCTGGAG CCAGAGACCGACACACGGGAGCCACTGACTCGGATCC GCAACAACTCAGCCATCCACATCCGAGTCTTCAGGGTC ACACCCAAGTAATTGAAAAGACACTCCTCCACTTATCCC CTCCGTGATATGGCTCTTCGCATGCTGAGTACTGGACCT CGGACCAGAGCCATGTAAGAAAAGGCCTGTTCCCTGGA AGCCCAAAGGACTCTGCATTGAGGGTGGGGGTAATTGT CTCTTGGTGGCCCAGTTAGTGGGCCTTCCTGA

Table 3 – bbCARMEN color code combinations

Color code	Dyes	AF546 nM	AF594 nM	AF647 nM
1	50 nM AF546; 0 nM AF594; 0 nM AF647	50	0	0
2	48.5 nM AF546; 0 nM AF594; 1.5 nM AF647	48.5	0	1.5
3	37.5 nM AF546; 0 nM AF594; 12.5 nM AF647	37.5	0	12.5
4	0 nM AF546; 50 nM AF594; 0 nM AF647	0	50	0
5	30 nM AF546; 20 nM AF594; 0nM AF647	30	20	0
6	47.5 nM AF546; 2.5 nM AF594; 0 nM AF647	47.5	2.5	0
7	0 nM AF546; 0 nM AF594; 50 nM AF647	0	0	50
8	0 nM AF546; 37.5 nM AF594; 12.5 nM AF647	0	37.5	12.5
9	0 nM AF546; 45 nM AF594; 5 nM AF647	0	45	5
10	0 nM AF546; 48.5 nM AF594; 1.5 nM AF647	0	48.5	1.5
11	45 nM AF546; 3.5 nM AF594; 1.5 nM AF647	45	3.5	1.5
12	35 nM AF546; 13.5 nM AF594; 1.5 nM AF647	35	13.5	1.5
